# Relevance of state–behaviour feedbacks for animal welfare

**DOI:** 10.1111/brv.70016

**Published:** 2025-03-24

**Authors:** Camille M. Montalcini, Charles C. Driver, Michael T. Mendl

**Affiliations:** ^1^ Department of Migration Max Planck Institute of Animal Behavior Am Obstberg 1 Radolfzell 78315 Germany; ^2^ Swiss Federal Institute for Forest Snow and Landscape Research Zürcherstrasse 111 Birmensdorf 8903 Switzerland; ^3^ Department of Psychology University of Zurich Binzmühlestrasse 14 Zürich Switzerland; ^4^ Bristol Veterinary School University of Bristol Langford House Langford BS40 5DU UK

**Keywords:** animal welfare, animal behaviour, feedback loops, tipping points, affective state, abnormal behaviours, dynamic models

## Abstract

An animal's behaviour and its state, including its health and affective state, are dynamic and tightly coupled, influencing each other over time. Although both are relevant to the animal's welfare, there has been limited research on their dynamics in welfare studies. Here we aim to: (*i*) review evidence for feedbacks between state and behaviour that could have beneficial or detrimental consequences for farm animal welfare; (*ii*) propose ways in which an understanding of such feedbacks could be used to enhance welfare; and (*iii*) provide practical guidance. We include as state variables any features that could influence the costs and benefits of an animal's behavioural actions, including individual characteristics and aspects of its social environment. We find evidence supporting positive state–behaviour feedback loops in various livestock species, suggesting that these loops could be common in farm settings and have significant welfare implications, such as leading to abnormal behaviours and persistent negative affective states. We suggest (*i*) estimating within‐individual feedback loops to extract individual characteristics for studying differences in welfare; (*ii*) identifying scenarios where change accelerated by positive feedbacks pushes an animal (or a group of animals) to a new state, also called tipping points; and (*iii*) generating positive feedback loops to elicit and maintain positive affective states. We end by encouraging use of dynamic models that integrate longitudinal data on animals' behaviour and state to enable exploration of their dynamics, and we provide a practical guide with annotated R code for support. Since the principles and ideas discussed here are relevant to any animals under human care, this approach could foster new perspectives for improving the welfare of all captive animals.

## I. INTRODUCTION

Positive feedbacks between an animal's state and its behaviour can explain how small differences between individuals, or even random fluctuations, can amplify over time and lead to consistent individual differences in state and behaviour (DeAngelis, Post & Travis, 1986; Sih *et al*., 2015). In behavioural ecology, these loops have attracted attention, especially through the lens of animal behaviour, helping to explain phenomena such as the enduring effects of early experiences on behaviour (Sih, 2011), apparent inconsistency between observed behavioural correlations (Dochtermann, 2023), and emergence of stable individual differences in behavioural tendencies (reviewed in Sih *et al*., 2015) and plasticity (Dingemanse & Wolf, 2013). However, it is important to recognise that these feedbacks can also be viewed from the perspective of an animal's state. For example, positive feedback loops that generate individual differences in behaviour would also lead to individual differences in state. While this aspect has been explored in ecological research, its relevance in settings where humans heavily influence animals' behaviour and state, such as farm environments, warrants further consideration. Here, after defining state–behaviour feedback loops, we review evidence for positive feedbacks between state and behaviour that could have beneficial or detrimental consequences for animal welfare. We then propose ways in which an understanding of these feedbacks can be used to enhance welfare. Finally, we provide a practical guide that discusses approaches for studying these loops, including a method that uses recently developed models, supported by annotated R code.

### (1) State–behaviour feedback loops

We here refer to a positive (or negative) feedback loop between a state *S* and behaviour *B*, when a change in *S* leads to a change in *B*, which in turn leads to a change in *S* in the same (or opposite) direction as the initial change in *S* (DeAngelis *et al*., 1986). In this context, the states of an individual are typically represented by any features that could influence the costs and benefits of its behavioural actions (Houston & McNamara, 1999; Sih *et al*., 2015). In line with this literature, we use the term ‘state’ in a technical sense, acknowledging that it can encompass individual traits, affective states, and other factors that shape behavioural decisions. More specifically, these states include characteristics of the focal individual, such as those linked to its morphology, physiology, social rank, affective state, knowledge about the environment, skill set, and aspects of its physical condition, such as its parasite load and its body temperature. Additionally, state variables also include characteristics of the focal individual's social environment, including the behaviours and health conditions of conspecifics.

**Fig. 1:**
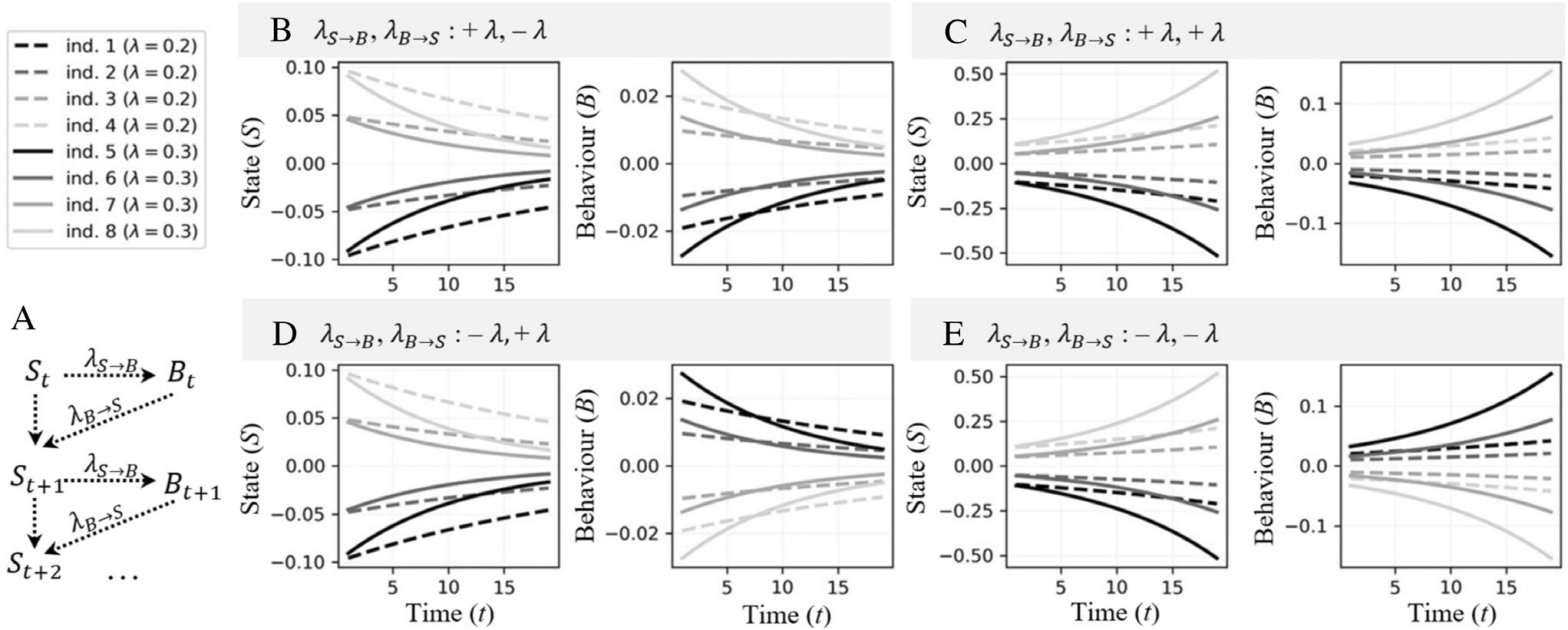
(A) Simple model structure representing a state–behaviour feedback loop, where an individual's current state $(S_{t}$) depends on its previous state ($S_{t-1}$) and previous behaviour ($B_{t-1}$): $S_{t}=S_{t-1}+\lambda_{B\to S}\cdot B_{t-1}+e_{t,S}$; and the current behaviour ($B_{t}$) depends only on the current state: $B_{t}=\lambda_{S\to B}\cdot S_{t}+e_{t,B}$. Residuals are represented by $e_{t,S}$ and $e_{t,B}$, but equal zero in the fig. (B–E) Illustration of this model for eight individuals each in four scenarios, described by the sign of the effects: (B) $\lambda_{S\to B}$, $\lambda_{B\to S}$: +,–; (C): $\lambda_{S\to B}$, $\lambda_{B\to S}$: +,+; (D): $\lambda_{S\to B}$, $\lambda_{B\to S}$: –,+; (E): $\lambda_{S\to B}$, $\lambda_{B\to S}$: –,–. Thus, negative feedback loops are illustrated in B and D, and positive ones in C and E. Individuals are uniquely determined by their associated $S_{0}$ (highlighted by different shades of grey and black) and magnitude of the effects (represented by the dashed and solid lines, for λ = 0.2 and λ = 0.3, respectively).

State–behaviour feedback loops can be represented with a simple model (Sih *et al*., 2015; Dochtermann, 2023), where an individual's current state depends on its previous state and previous behaviour, multiplied by a parameter $\lambda_{B\to S}$ determining the effect of *B* on *S*; and its current behaviour depends on its current state, multiplied by a parameter $\lambda_{S\to B}$ determining the effect of S on B (Fig. 1A). The magnitude and sign of a feedback loop are determined by multiplying the two effects: $\lambda_{S\to B}$ and $\lambda_{B\to S}$. A positive sign indicates a positive feedback loop (i.e. both effects are either positive or negative) and a negative sign indicates a negative feedback loop (i.e. when one effect is positive and the other negative). Figure 1B–E illustrates this simple model for eight individuals, each illustrating the four possible combination of signs [$\lambda_{S\to B}$, $\lambda_{B\to S}$: +,– (Fig. 1B); $\lambda_{S\to B}$, $\lambda_{B\to S}$: +,+ (Fig. 1C); $\lambda_{S\to B}$, $\lambda_{B\to S}$: –,+ (Fig. 1D); $\lambda_{S\to B}$, $\lambda_{B\to S}$: –,– (Fig. 1E)]. Negative feedbacks between a state and a behaviour typically promote their stability, leading to among‐individual convergence over time (Fig. 1B,D), whereas positive feedbacks typically promote among‐individual divergence over time (Fig. 1C,E) (DeAngelis *et al*., 1986; Sih *et al*., 2015).

## II. EVIDENCE FOR AND CONSEQUENCES OF POSITIVE FEEDBACKS RELATED TO ANIMAL WELFARE

Using examples from farm animals, Table 1 provides a non‐exhaustive list of potential state–behaviour feedback loops that could have consequences for animal welfare, and proposes associated putative mechanisms. These examples are further discussed within one of six subsections aimed at organising them into their potential welfare consequences: (*i*) prolonged positive or negative affective states; (*ii*) development of behavioural competence; (*iii*) prolonged positive or negative social experiences; (*iv*) increased physical impairments; (*v*) increased expression of abnormal and damaging behaviour; and (*vi*) increased parasite load (referred to as ‘Welfare consequence’ in Table 1). These loops could lead to individual (IW) or group (GW) differences in welfare, or to individual differences in a behaviour that could affect the welfare of conspecifics interacting with that individual (CW) (referred to as ‘Level’ in Table 1). We consider as state variables any features that can influence the costs and benefits of an individual's behavioural actions, including characteristics of the focal individual (section 1 of Table 1) and of its social environment (section 2 of Table 1) (Sih *et al*., 2015). We also expand these variables to include those relevant to a group of animals, i.e. considering the state and behaviour of a group rather than an individual (section 3 of Table 1), given the importance of social dynamics for captive animals.

**Table 1:** Examples of putative mechanisms that could generate feedbacks between state and behaviour in farm animals, with implications for their welfare. Welfare consequence categories are: (1) prolonged positive or negative affective states; (2) development of behavioural competence; (3) prolonged positive or negative social experiences; (4) increased physical impairments; (5) increased expression of abnormal and damaging behaviour; and (6) increased parasite load. The colour of these numbers indicates: positive consequences (green), positive or negative consequences (blue), negative consequences (red). These loops could lead to individual (IW) or group (GW) differences in welfare, or to individual differences in a behaviour that could affect the welfare of conspecifics interacting with that individual (CW), listed under Level. We use the notation *S* → *B* to refer to one side of the feedback loop, where a change in state *S* leads to a change in *B*, and *B* → *S* to refer to the other side, where a change in *B* leads to a change in *S*. When multiple feedback loops exist between two variables, comprising both positive and negative signs, the net sign of the feedback loop between the two variables will depend on the relative importance of each of these loops. Examples are discussed more fully in the text.

‐Welfare consequence	State ←→ Behaviour	Putative mechanism	Description and references
‐Level			
‐Example species			
Section 1: Characteristics of the focal individual as state variables			
Affective state			
(1) IW Various	Negative/positive moods ←→ ‘Pessimistic’/‘optimistic’ behaviour	Affective state‐mediated changes in decision‐making and attention themselves influence affective state	Mendl *et al*. (2010) suggested that animals in a negative mood could be caught in a positive feedback loop, whereby negative moods increase anticipation of negative situations and pessimistic interpretations of ambiguity, which in turn could decrease positive emotional experiences and thereby intensify the negative affective state. Similarly, animals in a positive mood would increase anticipation of positive situations and optimistic interpretations of ambiguity, which in turn could increase positive emotional experiences and thereby intensify the positive affective state.
(1) IW Horses, mink	Frustration‐like states ←→ Behavioural performance	Thwarting‐induced frustration‐like states impair skills which exacerbate further thwarting	A positive feedback loop between frustration‐like states (caused by thwarting of motivated behaviours) and behavioural performance has been suggested in certain domesticated animals, whereby higher frustration levels would reduce performance, which in turn would increase frustration [e.g. visible and invisible displacement task in horses (Rørvang *et al*., 2021); choice competition test in male mink (Díez‐León *et al*., 2013; Lewis *et al*., 2022)].
Learning state			
(2) IW – CW Various	Experiences with a behaviour ←→ Exhibiting that behaviour	Learning or skill improvement	As an individual gains experience exhibiting a behaviour, it tends to get better at performing the behaviour, making it advantageous to persist in that behaviour (Sih *et al*., 2019). This generates a positive feedback loop between a behaviour and an individual's experience with that behaviour.
(2) IW Various	Experience with a reinforcer ←→ ‘Desirable / trained behaviours’	Reinforcement learning	Positive experiences with humans achieved through rewarding animals after they exhibit desirable behaviour increase occurrences of desirable behaviours, which in turn increase rewards and positive experiences with humans [e.g. dairy cows (Honorato *et al*., 2012; Costa *et al*., 2021) and horses (Rochais *et al*., 2014)].
Hormonal state			
(3) IW, GW, CW Various	Oxytocin level ←→ Affiliative behaviour	Oxytocin‐mediated positive social behaviour leads to enhanced oxytocin levels in performers and recipients	Chen & Sato (2017) proposed that increased oxytocin concentration can induce positive social behaviour, which in turn may increase oxytocin concentration. This loop may also spread affiliative behaviour to the wider group for some period of time, as conspecifics involved in the behaviour may also undergo a similar loop.
(3) CW, GW Pigs	Testosterone level ←→ Agonistic behaviour – performance	Testosterone‐mediated aggression enhances testosterone levels	In pigs, aggressive behaviour may increase testosterone levels, which in turn could increase aggressiveness (Fredriksen *et al*., 2008), including in the wider group.
Health state			
(4) IW Rabbits, laying hens	Physical impairments ←→ Movement behaviours	Health problem impacts effective behaviour which exacerbates health problem	Positive feedback between physical impairments and movement behaviours can emerge if the physical impairments alter movements in a way that would in turn exacerbate physical impairments [e.g. spinal deformation – inactivity in rabbit does *B*➔*S* (Drescher & Loeffler, 1996), *S*←*B* (Carmel, 2013); keel bone fractures – hazardous landings in laying hens *S*➔*B* (Montalcini *et al*., 2024), *B*➔*S* (Sandilands *et al*., 2009; Stratmann *et al*., 2015a,b); keel bone fractures – time spent on perches in laying hens *S*➔*B* (Casey‐Trott & Widowski, 2016), *B*➔*S* (Appleby *et al*., 1993; Donaldson *et al*., 2012; Hester *et al*., 2013; Stratmann *et al*., 2015b)].
(6) IW Various	Parasite load ←→ Boldness and exploration	Parasite‐mediated changes in energy expenditure increase the need for resources, promoting behaviours that enhance parasite exposure	Parasite infections can elevate energetic needs and promote boldness and exploration to secure resources, which, in turn, can increase the risk of encountering parasites due to riskier or greater interactions with the physical and social environment (Barber & Dingemanse, 2010).
Motivational state			
(5) IW Pigs, broilers, horses	Motivation to perform goal‐oriented behaviour ←→ Consummatory behaviour	Inability to achieve feeding consummatory goals encourages further feeding‐related behaviour	Positive feedbacks between motivation to feed and feeding behaviour (Wiepkema, 1971), although initially beneficial, may eventually lead to abnormal behaviours when captive conditions prevent animals from achieving their consummatory goals (Mason & Mendl, 1997; Ijichi *et al*., 2013), as suggested in sows (Lawrence & Terlouw, 1993; Morgan *et al*., 1995; Haskell *et al*., 1996; Bergeron & Gonyou, 1997), broiler breeders (Savory *et al*., 1992; Lawrence & Terlouw, 1993), and horses (Roberts *et al*., 2017).
Section 2: Characteristics of the focal individual's social environment as state variables			
Conspecific health and welfare as state			
(5) CW Laying hens	Poor body condition of the recipient ←→ Agonistic behaviour – performance	Damaging behaviour causes damage that attracts further damaging behaviour	Damaging behaviour directed towards an individual can lead to a degradation of the individual's state in a way that would in turn increase the rate of damaging behaviours that it receives [e.g. severe feather pecking (Freire *et al*., 1999; McAdie & Keeling, 2000) and other injurious pecking causing bleeding as the presence of blood can attract hens to peck further (Duncan & Hughes, 1972)].
Conspecific behaviours as state			
(3) CW Various, sows	Conspecific aggression ←→ Agonistic behaviour – performance	Aggressiveness mediated by aggressiveness of conspecifics	There may be a positive feedback loop in which individuals exhibiting more aggressive behaviours are more likely to elicit aggressive behaviours in their conspecifics (McGlothlin *et al*., 2010) [e.g. in sows with bites, knocks, and pushes behaviours (Rault, 2016)].
Section 3: Characteristics of the focal group of animals as state variables			
Health / welfare state (of the focal group of animals)			
(5) GW Pigs, laying hens	Conspecific health and welfare ←→ Incidence of abnormal behaviours	Health or welfare problems in an animal can lead to the expression of damaging social behaviours, which in turn may also result in similar problems for the recipient	The proportion of individuals in a group with impaired health or welfare may influence the overall rate of damaging social behaviour which in turn can further impact group welfare [e.g. infection – tail biting in pigs (Boyle *et al*., 2022); infection – severe feather pecking in hens *S*➔*B* (Green *et al*., 2000; Parmentier *et al*., 2009; Nicol, 2019), *B*➔*S* (Nicol, 2019); stress‐level – piling in hens (Gray *et al*., 2020)].
(6) GW Various	Conspecific parasite load ←→ Social interactions	Parasite manipulation of host behaviour enhancing parasite spread within the group	Through parasite manipulation of host behaviour, feedback between parasite load in a group and social interactions could increase parasite transmission and load in the group (Ezenwa *et al*., 2016; Poulin, 2018; Hawley *et al*., 2021; Hawley & Ezenwa, 2022) [examples from behavioural ecology of behaviours related to social interactions that could increase in infected individuals: swarming propensity (Rode *et al*., 2013), time spent at feeders (Adelman *et al*., 2015), and reciprocal allogrooming in areas inaccessible *via* self‐grooming (Hawley & Ezenwa, 2022)].

### (1) Prolonged positive or negative affective states

#### (a) Feedbacks involving negative or positive moods

Affective states are characterised by two key dimensions: arousal (degree of activation) and valence (positivity *versus* negativity) (Mendl, Burman & Paul, 2010). These states may serve specific adaptive functions (Nesse & Ellsworth, 2009; Nettle & Bateson, 2012). For instance, when triggered by rewarding or punishing stimuli, affective states are thought to regulate approach and avoidance behaviours (Rolls, 2005; Burgdorf & Panksepp, 2006). More specifically, positive low‐arousal states (e.g. relaxed, calm) may aid in maintenance and recovery in low‐threat environments, while negative high‐arousal states (e.g. fearful, anxious) are linked to rapid avoidance responses in high‐threat environments (Mendl *et al*., 2010). Similarly, positive high‐arousal states (e.g. excited, happy) could facilitate the pursuit and acquisition of rewards in environments where resources are abundant, while negative low‐arousal states (e.g. sad, depressed) could support the conservation of energy in environments where resources are scarce (Mendl *et al*., 2010).

Although negative affective states may play an adaptive role in responding to challenges which should benefit animals' fitness in the natural environment (Nesse & Ellsworth, 2009), these benefits may be less evident in intensive settings, where some animals may instead become trapped in detrimental feedback loops that prolong their negative affective states. For example, fear is considered an adaptive state, with fear behaviours functioning to protect animals from predators and injuries. However, in intensive settings, certain fear responses, such as violent escape or panic, can be inappropriate and result in injury, pain or even death (Jones, 1996). Furthermore, in environments marked by frequent fear‐inducing events, an animal may enter an enduring negative state that increases its chances of interpreting events as dangerous and exhibiting associated avoidance behaviours, which in turn increases its experience of fear‐related states and reinforces its initial negative state. A related concept known as ‘fear generalisation’ posits that experiences increasing fear towards one danger would result in a parallel increase in fear towards other events (Sih *et al*., 2023). Sih (2011) emphasised the importance of stress carryovers, where the effects of a stressor (e.g. early social stress) may carry over to influence subsequent stress responses to other kind of stressor (e.g. response to danger). In this work, he suggested state–behaviour feedbacks as a framework to understand responses to stress better.

**Fig. 2:**
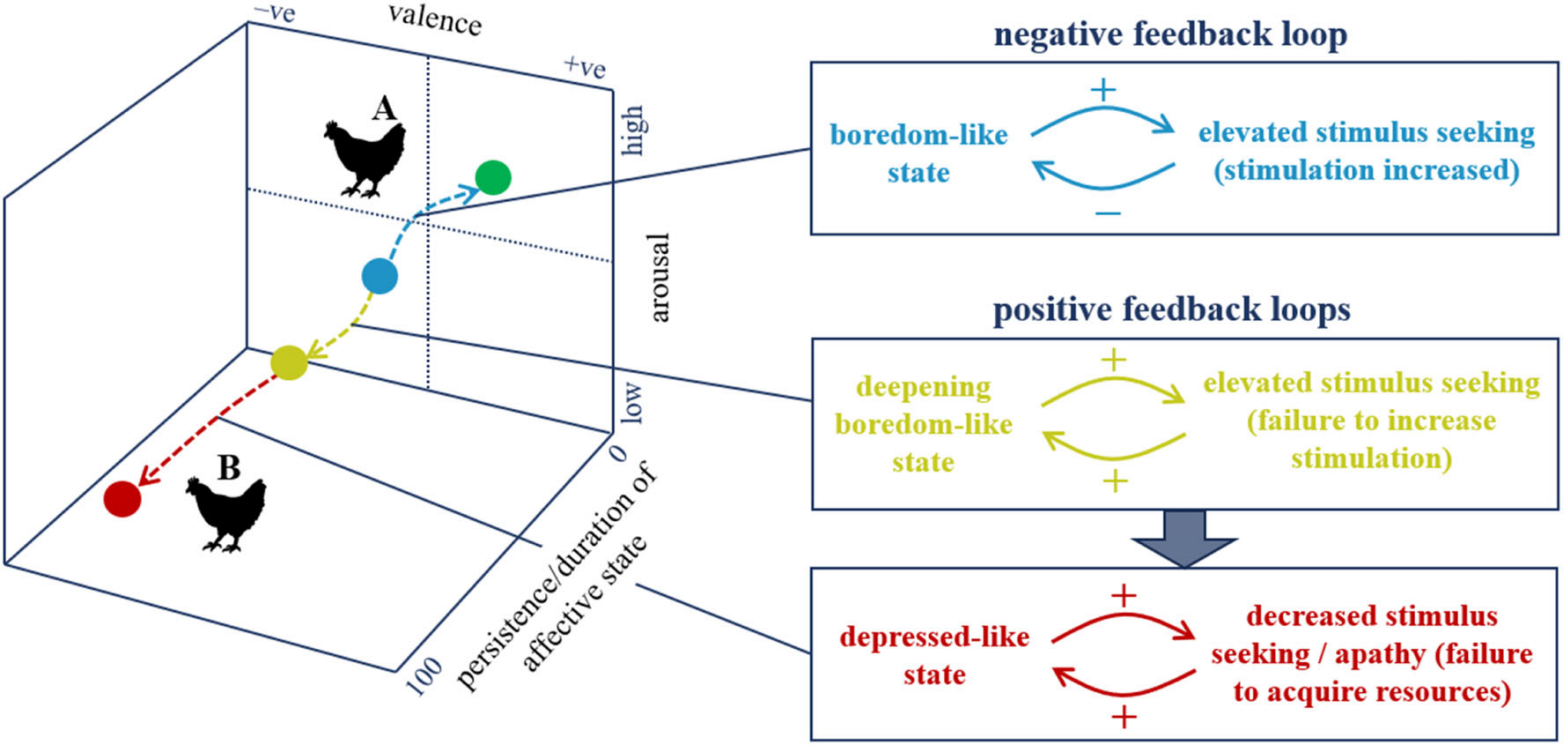
A three‐dimensional affective state space and a fictional example of feedback loops operating in sequence. The three‐dimensional space represents affective state in terms of valence, arousal and persistence in time [i.e. ranging from very short lived (~0) to very long lasting (~100)]. The dashed lines represent the fictional affective state trajectories of two hens (A and B) in this space, both starting in a short‐term boredom‐like state (blue circle) accompanied by increased stimulus seeking due to inadequate stimulation in their environments. For hen A, the behaviour is successful, stimulation is increased, and this results in a negative feedback loop between behaviour and state, leading to a more positive affective state (green circle). This transition is indicated by the blue dashed arrow. For hen B, the stimulus‐seeking strategy is unsuccessful and, over time, this leads to a deepening and more prolonged boredom‐like state (yellow circle) despite sustained stimulus seeking (which could develop into abnormal behaviour). This transition is illustrated by the yellow dashed arrow with the associated positive feedback loop also shown in yellow. Over time, stimulus‐seeking behaviour will likely gradually wane due to its continuing lack of success at increasing stimulation/acquiring reward, eventually resulting in a prolonged depressed‐like state (red circle) and associated apathetic‐like behaviour. These transitions are shown by the red dashed arrow in state space, and the thick blue arrow between positive feedback loops (see text for details).

It is possible that different loops involving similar states and behaviours act in sequence to explain the emergence of long‐term affective states. However, the sequences in which these different loops may operate remain unclear and likely differ across individuals and environments. A fictional example of such a sequence is illustrated for an initial boredom‐like state and its development across time in Fig. 2. This figure presents a three‐dimensional space composed of valence, arousal and a scale of ‘persistence/duration’, indicating the degree to which an affective state persists over time. Behaviours associated with boredom‐like affective states, such as those reflecting increased interest in and seeking of stimuli, like exploration and increased responsiveness (Meagher & Mason, 2012), could provide adaptive benefits in conditions where animals can explore new environments or engage with various stimuli. This might occur in natural settings or when ‘bored’ farm animals are transferred to new environments. In these cases, enhanced stimulus‐seeking associated with ‘boredom’ could increase environmental stimulation experienced by the animal, which would then reduce the boredom‐like state, generating a more positive affective state. This negative feedback loop is indicated in blue in Fig. 2. However, in situations where captivity prevents animals from encountering novel environments or stimuli, enhanced stimulus‐seeking might instead result in many unrewarded monotonous experiences which in turn reinforce the initial state and associated behaviours leading to a deepening boredom‐like state. This positive feedback loop is indicated in yellow in Fig. 2. With time, stimulus‐seeking may wane due to lack of success of this behavioural strategy at increasing stimulation and rewarding events, eventually resulting in a negative depression‐like affective state and associated apathetic‐like behaviour. This transition is indicated by the thick blue arrow between the positive feedback loops in Fig. 2. A further positive feedback loop shown in red in Fig. 2 would act to sustain the depression‐like state. However, as this apathetic‐like ‘withdrawal’ behaviour may also reduce negative experiences, the impact on the hen's state will depend on the balance of positive and negative events in the environment.

More generally, longer‐term affective states, including bouts of anxiety and depression, also referred to as mood, may reflect cumulative shorter‐term affective experiences, like fear and sadness, and shape expectation for current and future decisions (Mendl & Paul, 2020). Negative mood states could increase anticipation of negative situations and pessimistic interpretation of ambiguity leading to increased pessimistic behaviours, which could decrease positive experiences and further shift the balance of experience towards a more negative state (Mendl *et al*., 2010).

Feedbacks between positive moods and optimistic behaviours could also occur and, given the absence of some factors that may lead to more negative affective states in the natural environment (e.g. predators or absence of food), they have the potential to be more persistent in captive settings than in nature. Specifically, in environments with low level of threats and frequent food treats or other rewarding events, animals in a positive mood may increase anticipation of positive situations, show optimistic decision‐making under ambiguity that likely leads to positive outcomes, hence increasing positive short‐term emotional experiences and thereby intensifying their long‐term positive affective state (Mendl *et al*., 2010).

The dynamics between positive moods and optimistic behaviours may differ from those occurring between negative moods and pessimistic behaviours. This difference could be due to the high cost of a false negative (i.e. failing to detect a true threat) that contrasts with the low cost of a false positive (i.e. false alarm) when detecting threatening cues, whereas the costs of false negatives and false positives may generally be more comparable when detecting positive cues. The preponderance of false alarms exhibited by defence regulation systems occurring in environments where threats can exist, has been referred to as ‘the smoke detector principle’ (Nesse, 2005). Thus, animals in a negative affective state induced by frequent threat experiences may rapidly exhibit pessimistic behaviour (and hence increase occurrences of false alarms) to enhance their chances of detecting all threats. By contrast, animals in a positive affective state induced by frequent positive experiences may more gradually exhibit optimistic behaviours. In other words, ‘negative mood – pessimistic behaviour’ feedback loops could, overall, be of greater magnitude than ‘positive mood – optimistic behaviour’ feedback loops when examined across a range of contexts relevant to farm settings. As a result, farm animals could generally be more prone to persistent pessimistic behaviours than optimistic ones within short time intervals. Simulation‐based studies should, however, test this idea and investigate how the dynamics between moods and behaviours vary with individual traits and experiences across different contexts.

Despite theoretical support for mood–behaviour feedback loops relevant to farm animal welfare, no empirical study has concurrently examined the dynamics of the two components of these loops, probably because it remains challenging to repeatedly measure both components independently. Typically, pessimistic/optimistic behaviours, measured *via* judgment bias tests, have served as a proxy indicator of affective states, since subjective states are impossible to measure directly. Many studies have manipulated animals' affective state with pharmacological and non‐pharmaceutical drugs and have generally found that the assumed change in affective state altered judgement bias as expected [for meta‐analyses, see Neville *et al*. (2020b) and Lagisz *et al*. (2020)]. Conversely, there have been studies indicating that stable individual differences in the propensity to exhibit optimistic or pessimistic responses under ambiguity can influence the ability of animals to deal with challenge and hence their resulting affective responses to welfare threats (in rats; Rygula, Papciak & Popik, 2013). However, to our knowledge, no empirical studies in farm animals have explored the dynamics of the interplay between optimistic/pessimistic behaviours and affective states, or how this could vary across individuals (e.g. due to differences in memorising positive *versus* negative events) and environments (e.g. level of threats relative to threat cues). Theoretical, and potentially empirical, investigations of feedbacks between affective states and behaviours could enhance our understanding of the mechanisms underpinning long‐term affective states. For example, recent studies of human judgment biases have used computational modelling to assess the short‐term interplay between in‐task reported affective states and decision‐making under ambiguity. In these early studies, momentary negative affect has been associated with increased risk‐seeking decisions whilst positive affect is associated with periods when more positive reward prediction errors occur and things are going better than expected (e.g. Rutledge *et al*., 2014; Neville *et al*., 2021). Such computational approaches are also being trialled in laboratory animal studies (Neville *et al*., 2020a), although measures of in‐task affective state remain challenging to obtain.

Finally, it is important to note that, while these positive feedback loops may exist in farm settings, negative feedback loops could prevent their development. For example, the prediction error generated by an animal in a negative state encountering a positive situation (or *vice versa*) may lead to a strong positive response that would reduce subsequent pessimistic behaviours (e.g. Rutledge *et al*., 2014; Eldar *et al*., 2016).

#### (b) Feedbacks involving frustration‐like states

A frustration‐like state may occur when an animal fails to attain an expected outcome. This negative state, in turn, may reduce the animal's ability successfully to perform behaviours required to achieve expected outcomes. A positive feedback loop between the level of frustration and task performance has been suggested in certain domesticated animals. For example, it was shown that highly stereotypic male mink mated less often in choice competition tests, indicating impaired social competence or attractiveness in individuals performing more stereotypic behaviours, like pacing (Díez‐León *et al*., 2013). Lewis *et al*. (2022) suggested that this result could indicate a positive feedback loop whereby mating frustration led to the development of stereotypic behaviours that reduced mating performance which in turn increased frustration. Similarly, horses that were unsuccessful in completing visible and invisible displacement tasks had higher heart rate levels and exhibited more behaviour indicative of frustration (Rørvang *et al*., 2021). The authors suggested that this could in turn have hampered performance in subsequent trials. As tasks that are too challenging may lead to frustration‐like states, whereas too‐simple tasks may lead to boredom‐like emotions (Csikszentmihalyi, 1990), this detrimental loop underscores the importance of providing farm animals with tasks fitting individuals' cognitive abilities.

### (2) Development of behavioural competence

State–behaviour feedback loops could be a mechanism enabling the development of behavioural competence, an aspect related to positive animal welfare, which has been recently defined as ‘the animal flourishing through the experience of predominantly positive mental states and the development of competence and resilience’ (Rault *et al*., 2025, p. 2). Here, we will briefly discuss behaviour–experience feedback loops that typically occur naturally and those that may be induced to achieve desirable, trained behaviours.

Typically, as an individual gains experience exhibiting a behaviour, it tends to get better at performing the behaviour (i.e. develops behavioural competence), making it advantageous to persist in that behaviour (Sih, Sinn & Patricelli, 2019). This generates a positive feedback loop between a behaviour and an individual's experience with that behaviour, which can lead to individual differences in behavioural competence. Similar loops can be initiated by animal caretakers to achieve desirable behaviours. For example, the use of positive reinforcements like tactile contact and feeding in order to improve human–animal relationships fits this feedback loop framework [e.g. dairy cows (Honorato *et al*., 2012; Costa *et al*., 2021) and horses (Rochais *et al*., 2014)]; rewarding animals after desirable behaviours increases occurrences of those behaviours which in turn increases rewards and positive experiences. Future studies could investigate how reinforcers distributed by automated systems within farm settings could enhance the development of desirable behavioural competences.

### (3) Prolonged positive or negative social experiences

State–behaviour feedback loops can also prolong positive or negative social experiences. For example, Chen & Sato (2017) proposed that increased oxytocin concentration could induce positive social behaviour, which in turn could increase oxytocin concentration. Fredriksen *et al*. (2008) similarly suggested that in farmed pigs, aggressive behaviour could increase testosterone levels, which in turn might increase aggressiveness.

In addition to individual hormone levels, the state of an individual's social environment could also contribute to reinforcing positive or negative social experiences. McGlothlin *et al*. (2010) proposed that there could be a positive feedback loop in which individuals exhibiting more aggressive behaviours are more likely to elicit aggressive behaviours in their conspecifics. This dynamic could be linked to testosterone‐mediated aggressiveness feedback or to an individual fighting back and persisting in the behaviour as it becomes more competent, among other possibilities. Rault (2016) found positive feedback loops in sows kept at high stocking density; bites encouraged bites in return, and knocks or pushes encouraged knocks or pushes in return, thus elevating group‐level aggression with potential impacts on conspecific welfare. These feedback loops could be further explored in relation to stocking density to determine whether there is a stocking density threshold beyond which the spread of aggressive behaviours becomes significantly more likely. These feedbacks may also spread affiliative behaviours, and ultimately lead to some groups being consistently more aggressive or affiliative than others.

### (4) Increased physical impairments

Positive feedback between physical impairments and movement behaviours can emerge if the physical impairments alter movements in a way that would in turn exacerbate physical impairments, for example by increasing the animal's vulnerability to (further) injury, or by preventing it from accessing resources that may help to alleviate the impairment. While this section focuses on three potential detrimental feedback loops between physical impairments and movement behaviours in rabbits or laying hens, it is possible that common impairments in other farmed species, such as lameness in cattle, sheep or pigs, induce similar effects.

In captive conditions where animals may struggle to achieve the necessary activity levels to maintain healthy bones, inactivity can increase bone fragility and thereby susceptibility to damage (Marchant & Broom, 1996). In turn, such damage may further increase inactivity. For instance, in reproductive does (i.e. the female parent stock of meat rabbits) housed in small individual wire cages, spinal deformations are thought to result from bone degradation due to inactivity (Drescher & Loeffler, 1996), and in turn are thought to reduce the animal's mobility (Carmel, 2013). Also, increased inactivity can reduce leg bone quality in rabbit does (Buijs *et al*., 2014), which in turn could increase lying time and thereby inactivity. As the influence of activity on bone density and strength diminishes when animals transition from juveniles to adults (Pearson & Lieberman, 2004), such loops may be more common in younger animals.

In commercial laying hens, keel bone fractures (KBFs) are particularly prevalent in complex three‐dimensional systems like multi‐tier aviaries, where failed landings are considered one potential factor increasing the severity or prevalence of KBF (Sandilands, Moinard & Sparks, 2009; Wilkins *et al*., 2011; Stratmann *et al*., 2015a,b). Conversely, more severe KBFs were shown to predict later increase in the number of tiers crossed while flying or jumping between two of the aviary tiers (Montalcini *et al*., 2024). Given that increased height could be a risk factor for failed landings, this result suggests that hens with more severe KBF may be at a greater risk of failed landings. Therefore, hens with KBF that do not sufficiently reduce movements between aviary tiers could have a higher incidence of failed landings which in turn leads to more severe KBF.

Time spent on perches and severity of KBF may also be involved in a detrimental positive feedback loop. The presence of perches has been shown to increase the prevalence of keel bone damage, including deviations of the bone and fractures (Appleby, Smith & Hughes, 1993; Donaldson, Ball & O'Connell, 2012; Hester *et al*., 2013). It is generally thought that high‐energy collisions with perches may lead to or aggravate existing fractures, while time spent on perches may increase bone deviations (Stratmann *et al*., 2015b). However, in conventional cage systems, perches have been shown to increase fracture prevalence (Abrahamsson & Tauson, 1993; Abrahamsson, Tauson & Appleby, 1996; Hester *et al*., 2013), although it is unlikely that collisions are responsible for the fractures in these systems. Furthermore, given that the keel is the main support for body weight during perching (Pickel, Schrader & Scholz, 2011) and the associated pressure may result in bone deviations (Stratmann *et al*., 2015b), it is also plausible that increased time spent on perches increases the severity of existing fractures. Conversely, it has been shown that hens with multiple fractures perch longer than hens with no fractures (Casey‐Trott & Widowski, 2016) (with an opposite trend in housing systems where birds needed to fly to access perches; Nasr *et al*., 2012), possibly indicating a strategy to reduce interactions with conspecifics or to seek refuge to avoid predators. Yet, it is also possible that these behavioural differences were exhibited before the fracture, and future research is needed to test whether more severe KBFs can increase perch use and whether this in turn can increase KBF severity.

### (5) Increased expression of abnormal and damaging behaviour

**Fig. 3:**
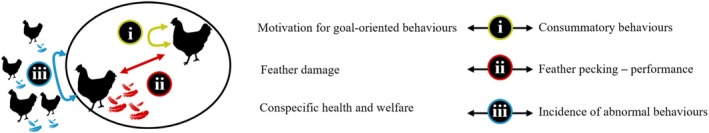
Three potential feedback loops illustrating how multiple loops operating at different levels could simultaneously contribute to the spread of damaging behaviour in laying hens. The figure illustrates loops within an individual (i), between two individuals (ii), and within a group of animals (iii). The loops are described on the right side of the figure, with state variables positioned on the left side of the arrows and behaviours on the right.

Abnormal behaviour can be defined as ‘behaviour which differs in pattern, frequency or context from that which is shown by most members of the species in conditions that allow a full range of behaviour’ (Broom, 2019, p. 1). Some of these abnormal behaviours can be damaging, such as feather pecking in laying hens and tail biting in pigs (Brunberg *et al*., 2016); both can escalate into cannibalism, causing severe wounds or death. Abnormal and damaging behaviours are frequent in farm animals (Lambton *et al*., 2010; Lauber *et al*., 2012; Roberts *et al*., 2017) and can be a sign of reduced welfare (Mason & Latham, 2004). Repetitive abnormal behaviours generally fall into two classes: behaviours derived from repeated goal‐oriented actions, called impulsive or compulsive behaviours, and behaviours derived from repeated motor function, called stereotypic behaviours (Ridley, 1994; Garner, 2005). In this section, we discuss three potential feedback loops, operating through different mechanisms, that may lead to repetitive and other abnormal or damaging behaviours as illustrated in Fig. 3, with examples provided for laying hens.

#### (a) Abnormal behaviour arises from feedback loops when consummatory goals are unfulfilled

Positive feedback loops between motivation to feed and feeding behaviour (Wiepkema, 1971), although initially beneficial, may eventually lead to abnormal behaviours when captive conditions prevent animals from achieving their consummatory goals (Mason & Mendl, 1997; Ijichi, Collins & Elwood, 2013). There appears to be a positive feedback loop that, following the onset of feeding behaviour, momentarily increases feeding motivation which stimulates the animal to feed, thereby increasing food intake and meal duration (Wiepkema, 1971). Once the consummatory goal (a state of satiation) is achieved, feeding behaviours no longer seem to boost feeding motivation, hence initiating a negative feedback loop that inhibits further feeding behaviour (Wiepkema, 1971; Hughes & Duncan, 1988). However, when animals are repeatedly unable to achieve consummatory goals and cannot redirect appetitive behaviours to other appropriate resources (e.g. food‐restricted livestock, such as broiler breeders and pregnant sows who cannot move elsewhere to forage), then the negative feedback on the motivation to feed does not occur and animals become locked into a positive feedback loop, potentially fuelled by dopamine increasing sensitivity to stimuli and motivation (Ijichi *et al*., 2013). As a result, some animals may develop stereotypic behaviours (Hughes & Duncan, 1988; Mason & Mendl, 1997; Ijichi *et al*., 2013), as suggested for sows (Lawrence & Terlouw, 1993; Morgan *et al*., 1995; Haskell *et al*., 1996; Bergeron & Gonyou, 1997), broiler breeders (Savory, Seawright & Watson, 1992; Lawrence & Terlouw, 1993), and horses (Roberts *et al*., 2017). It is possible that these stereotypic behaviours have dearousing properties that suppress the positive feedback loop (Savory *et al*., 1992; Lebelt, Zanella & Unshelm, 1998). Evidence for the link between attainment of consummatory goals and repetitive behaviour was provided by Bergeron & Gonyou (1997) who showed that sows fed with a high‐energy diet reduced repetitive chain manipulating and vacuum chewing compared to sows on a standard diet, thus indicating that increased energy provision could decrease the occurrence of stereotypies in pregnant sows.

This short‐term positive feedback loop between appetitive and consummatory behaviours that becomes negative as consummatory goals are reached, initially proposed by Hughes & Duncan (1988), may also partially explain the occurrence of feather pecking in chickens and tail biting in pigs (Bracke *et al*., 2018). Specifically, when animals lack sufficient foraging opportunities to fulfil their consummatory goals, their motivation to forage and persistent unsuccessful attempts at foraging behaviour could mutually reinforce each other and lead to redirection of foraging to other available substrates. Feather pecking in laying hens and tail biting in pigs are thought to be misdirected, or redirected, foraging behaviours (Huber‐Eicher & Wechsler, 1998; Grandin & Deesing, 2022). Empirical studies on domestic chickens indicated that housing conditions encouraging foraging behaviour can be effective in mitigating and preventing feather pecking (Huber‐Eicher & Wechsler, 1997, 1998; Wechsler & Huber‐Eicher, 1998; Aerni, El‐Lethey & Wechsler, 2000), although the impact of adding forage remains unclear, probably because feather pecking is a multi‐factorial problem (Cronin & Glatz, 2020). Future empirical work is required to confirm whether an animals' appetitive motivation to forage coupled with unsuccessful attempts at foraging mutually reinforce each other, and eventually result in redirected abnormal behaviours.

#### (b) Damaging behaviour causes damage triggering further damaging behaviour

In laying hens, damaging behaviour directed towards an individual can lead to a degradation of the individual's state in a way that would in turn increase the rate of damaging behaviours that it receives. For example, McAdie & Keeling (2000) experimentally manipulated feathers by damaging them at different degrees of severity and tested whether damaged feathers were pecked more than undamaged ones. Their results showed that more severe pecking occurred on damaged feathers than undamaged ones. Freire, Walker & Nicol (1999) also suggested that feather damage promotes feather pecking. He proposed that this behaviour can trap animals in a positive feedback loop where increased feather damage increases the rate of severe feather pecking directed towards that animal, which in turn further damages its feathers. It is possible that feedback loops with a similar mechanism apply to victims of other types of pecking, such as toe pecking, as the presence of blood can attract hens to peck more (Duncan & Hughes, 1972).

#### (c) Health and welfare status both drive and result from damaging social behaviours

There may also be positive feedbacks between the proportion of individuals with poor welfare in a group and the incidence of damaging social behaviours. For example, in farmed pigs, tail biting can lead to lesions that provide entry points for pathogens which may cause infections. These infections may adversely impact nutrient ingestion and processing, leading to increased nutrient deficiencies, which in turn, may increase the risk of tail biting by the infected individual (Boyle *et al*., 2022). Consequently, there may be a positive feedback loop between the number of infected pigs and the incidence of tail biting in a group. However, more evidence is needed for this feedback loop and whether it applies to other abnormal behaviours in pigs (Boyle *et al*., 2022).

Similarly, in laying hens there may be a positive feedback loop between the proportion of infected hens and the incidence of severe feather pecking. Occurences of egg peritonitis, infectious bronchitis (typically caused by *Escherichia coli*), and mites were found to be associated with feather pecking (Green *et al*., 2000; Heerkens *et al*., 2015). More precisely, severe pecking may increase susceptibility to infection by providing entry points for pathogens (Nicol, 2019). For evidence concerning the other direction of the feedback loop, it is thought that red mite may trigger injurious pecking (Niekerk, 2019; Nicol, 2019) and outbreaks of severe feather pecking have been reported during or after infectious outbreak, such as *E. coli* infections or chronic enteritis (Brunberg *et al*., 2016). There is also evidence indicating that the immune system might influence the emergence of feather pecking (reviewed in Michel *et al*., 2022). For example, Parmentier *et al*. (2009) found that inducing specific immunity in young chickens, similar to vaccination protocols on commercial farms, increased their likelihood of developing severe feather damage later in life compared to untreated animals, suggesting that immune responses induced by infections may predispose hens for feather pecking.

Positive feedbacks may also play a role in piling behaviour in which hens aggregate in densely packed groups, potentially resulting in smothering and mortality (Gray *et al*., 2020). Participation in piling behaviour can increase behavioural and physiological stress and arousal levels, which could in turn predispose further piling behaviour (Gray *et al*., 2020). However, future research is required to clarify whether any resulting behavioural or physiological stress actually increases or decreases incidence of piling (Gray *et al*., 2020). If it does increase, further work could also explore the existence of a broader feedback loop between indicators of stress at the group level and the incidence of other undesirable behaviours, including severe feather pecking and cannibalism. It seems plausible that these behaviours would induce stress in the recipients, and it has been hypothesised that stress may enhance the development of feather pecking (El‐Lethey *et al*., 2000; Louton *et al*., 2017) and of cannibalistic behaviour (Yngvesson & Keeling, 2001).

### (6) Increased parasite load

Behavioural change due to parasite infection could arise due to changes in energy expenditure and generate positive feedback loops that further increase parasite load (Sih *et al*., 2015). Parasite infections can elevate energetic needs and thus promote greater levels of boldness and exploration to secure resources, which in turn can increase parasite exposure through riskier or greater interactions with the physical and social environment (Barber & Dingemanse, 2010). For example, parasites in herbivores like sheep, cattle, goats, and horses may affect their grazing behaviour, making them less selective in their diet and thus to graze more near faeces containing other parasites (Hutchings *et al*., 1998). However, to our knowledge no empirical evidence has yet supported this detrimental positive feedback loop in farm animals. Instead, a study has supported an alternative hypothesis suggesting that parasites could induce anorexia to reduce feeding motivation despite the increased nutrient demand, so that parasitised animals would become more selective in their diet to reduce further parasite intake (Kyriazakis, Tolkamp & Hutchings, 1998). Empirical support for this hypothesis comes from a study showing that parasitised sheep exhibited a greater tendency to reject contaminated swards, were more selective against faecal contaminated swards, and decreased bite rates and depths, compared to non‐parasitised sheep (Hutchings *et al*., 1998). However, their results also showed that, compared to uninfected sheep, parasitised sheep more strongly avoided sward contaminated with infectious fresh faeces, likely due to their odour, resulting in a greater proportional level of contact with older faeces which pose a higher risk of parasitism compared to fresher ones. Therefore, future studies investigating the dynamics between grazing behaviour and parasite load should consider the important role of odours.

Through parasite manipulation of host behaviour, feedback between parasite load in a group and behaviours shaping characteristics of the social network could increase group parasite load (Ezenwa *et al*., 2016; Poulin, 2018; Hawley *et al*., 2021; Hawley & Ezenwa, 2022). Although infected individuals may isolate themselves from uninfected group members limiting spread of the parasite (Poulin, 2018), parasites can also induce their hosts to form groups (Rode *et al*., 2013), spend more time at feeders (Adelman *et al*., 2015), or encourage reciprocal allogrooming in areas inaccessible *via* self‐grooming (Hawley & Ezenwa, 2022), which could enhance parasite transmission. In fact, it is well established that many parasites can manipulate their hosts' behaviour as an adaptive strategy to enhance transmission (Moore, 2002; Thomas *et al*., 2005; Poulin, 2010). Interestingly, by affecting the “phylogenetically primitive structures of the limbic system and related neurochemical systems”, Klein (2003, p. 441) proposed that the effects of parasites on social behaviour may persist across multiple classes of vertebrates.

To our knowledge, the exploration of feedback between parasite infection and host behavioural change in farm animals has been relatively limited compared to that in wild animals. Yet, it could be relevant in animals with access to grazing pasture, in aquaculture (Bui *et al*., 2019), and in social animals. For example, experimental studies could assess how characteristics of a social network (like its modularity) prior to infection may influence the rate of infection spread and how this in turn impacts these characteristics. Implementing an anti‐parasite treatment would then allow investigation of whether the network reverts to its original state after the parasites are removed. Empirical studies of animal personality could also determine if boldness influences parasite risk in uninfected hosts, and if boldness persists in infected animals and further increases parasite load. Exploring the existence of these loops within commercially relevant settings and whether their magnitude is greater in animals in poor condition (Beldomenico & Begon, 2010) could also be interesting avenues for future research.

## III. POTENTIAL OF FEEDBACK LOOPS TO ENHANCE ANIMAL WELFARE

Having seen that a variety of welfare problems may be framed as resulting from feedback loops between state and behaviour, we now propose potential perspectives for studying these feedbacks ultimately to foster new avenues for improving animal welfare. Specifically, we suggest (*i*) estimating feedback loops at the individual level (i.e. within‐individual feedback loops) to extract individual characteristics for studying individual differences in welfare, (*ii*) identifying scenarios where change accelerated by positive feedbacks pushes an animal (society) system to new states, and (*iii*) generating positive feedback loops to elicit and maintain positive affective states.

### (1) Estimating within‐individual feedback loops to extract individual characteristics

We propose that characteristics of within‐individual state–behaviour feedback loops may be relevant as individual characteristics to study individual differences in welfare. That is, in addition to individual differences in state and behaviour, their dynamics may provide complementary information. Specifically, because positive feedback loops may have consequences that are not immediately apparent, two individuals exhibiting similar state and behaviour at a certain point in time, may, due to different state–behaviour feedbacks, ultimately show substantial differences in state and behaviour later on. For example, differences in the sign of feedback loops between physical impairments and space‐use behaviours could explain differences in persistence or severity of impairments. Considering the potential feedbacks between bone damage and perching behaviour, some hens might exhibit a positive feedback loop ($\lambda_{S\to B}$: +, $\lambda_{B\to S}$: +) while others a negative one ($\lambda_{S\to B}$: –, $\lambda_{B\to S}$: +), indicating different behavioural strategies in response to bone damage. The former hens would increase perch use to, for instance, avoid dominant birds (Cordiner & Savory, 2001), leading to more persistent damage. By contrast, the latter would reduce perch use due to, for instance, greater sensitivity to pain, and hence facilitate healing. Also, differences in the magnitude of feedback loops between negative mood and pessimistic behaviours, perhaps related to individual differences in reward and punishment sensitivity (e.g. Corr, 2004), could explain differences in individuals' susceptibility to persistent negative affective states.

One practical significance of quantifying individual differences in farm animals is to identify phenotypes, such as behavioural tendencies, that could be integrated into breeding practices to breed for more robust animals (Koolhaas & van Reenen, 2016). Examples from studies on farm animals extracting individual characteristics based on model‐derived estimates which have shown some level of heritability are individual estimates of behavioural plasticity and predictability (Martin *et al*., 2017; Prentice *et al*., 2020; Montalcini *et al*., 2023). We here suggest that studying within‐individual feedback loops may present new opportunities to improve breeding practices, provided that underlying characteristics are heritable and associated with welfare.

### (2) Identifying tipping points for early warning systems

#### (a) The notion of tipping points

van Nes *et al*. (2016, p. 904) defined the concept of ‘tipping point’ as ‘any situation where accelerating change caused by a positive feedback drives the system to a new state’. For example, social tipping points in animal society system have been referred to when the characteristics of a group undergo an abrupt transition in state (Pruitt *et al*., 2018), such as shifting from calm to violent states in response to heat stress (Doering *et al*., 2018). However, although an animal itself can be regarded as a biological system (Alcocer‐Cuarón, Rivera & Castaño, 2014) and could exist in different states, the concept of a tipping point, to our knowledge, has not yet been explored in situations where accelerating change caused by a positive feedback drives an individual to a new state. The evidence of positive feedback loops reported in this review, at both the group and individual levels, underscores the relevance of the concept of ‘tipping point’ in animal systems and provides concrete examples for plausible future exploration in both animal and social systems.

**Fig. 4:**
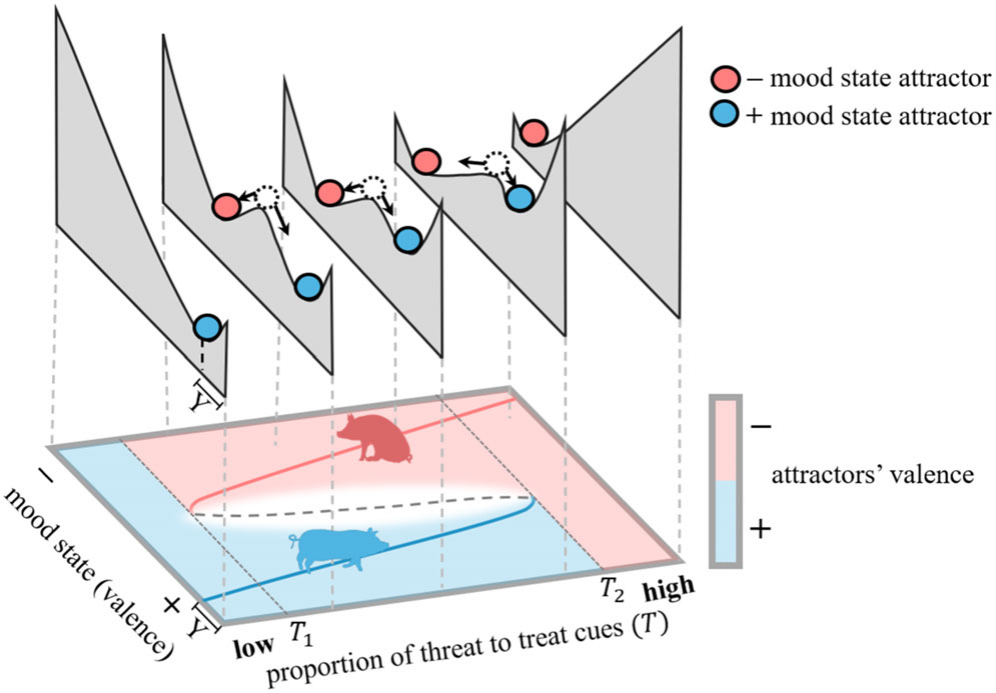
Figure from Pruitt *et al*. (2018), with some changes, published by the Royal Society under CC‐BY 4.0 license [https://creativecommons.org/licenses/by/4.0/], exemplifying an animal system in two different scenarios resulting in two different tipping points: (1) $T_{1}$: when the red pig is in a negative mood state for a given environmental condition (T > $T_{1}$) and the level of threat cues is decreasing relative to treat cues, it will eventually reach tipping point $T_{1}$, at which it transitions to a positive mood, and (2) $T_{2}$: when the blue pig is in a positive mood state for a given environmental condition (T < $T_{2}$) and the proportion of threat cues is increasing relative to treat cues, it will eventually reach tipping point $T_{2}$, at which it transitions to a negative mood. More generally, the states towards which an animal system tends to converge can be called ‘attractors’ and are represented by red and blue balls in the upper panel for the negative and positive mood states, respectively, as well as by the solid lines in the lower panel. The valence of these two attractors is represented by colours in the lower panel, for different states and environmental conditions. Deeper and steeper wells indicate a quicker return to the attractor state and greater resistance to noise. The deepest and steepest wells occur at the extremes ends of environmental conditions, when even relatively large changes in state do not affect the state in the long term. At intermediate environmental conditions, there are two attractors with an unstable equilibrium in between (illustrated with the dashed ball in the upper panel and dashed lines in the lower panel), where small changes in state or environment can drive the system to a new stable state.

The states towards which a system tends to converge given starting and current conditions can be called ‘attractors’. Figure 4 (adapted from Pruitt *et al*., 2018) shows a fictional example of alterations in the geometry of two ‘attractors’ as environmental conditions change in an animal system, for two different scenarios (i.e. a red and a blue pig with different starting conditions). Environmental conditions (T) are here represented by the proportion of threat cues relative to treat cues. These attractors are represented at the bottom of wells with red and blue balls, symbolising a negative and a positive mood state, respectively. At very low or very high proportions of threat cues relative to treat cues, there is only one attractor, whereas at intermediate environmental conditions there are two attractors. The walls of the wells define the strength of the positive feedback loops that maintain the system in a particular state. Specifically, the steepness and depth indicate how fast the system returns to the attractor state (i.e. stable equilibrium, represented by the solid lines in the lower visual) in response to noise. The deepest and steepest wells occur at the extremes ends of environmental conditions and indicate a quicker return to the attractor state and thus greater resistance to noise, i.e. even a relatively large change in state can dissipate and have no long‐term effect. Tipping points occur when these stable equilibria collide with an unstable equilibrium (represented by the peaks in between the two attractors in the upper visuals and by a dashed line in the lower visual). Figure 4 illustrates an animal system in two different scenarios that results in two different tipping points, $T_{1}$ and $T_{2}$. First, when the red pig is in a negative mood state for a given environmental condition (T > $T_{1}$) and the level of threat cues is decreasing relative to treat cues, it will eventually reach a tipping point $T_{1}$, at which it transitions to a positive mood. Second, when the blue pig is in a positive mood state for a given environmental condition (T < $T_{2}$) and the proportion of threatening cues is increasing relative to treat cues, it will eventually reach a tipping point $T_{2}$, at which it transitions to a negative mood. In this example, at intermediate environmental conditions ($T_{1}<T<T_{2}$), an animal can be either in a positive or a negative state depending on its past states.

From a more practical perspective, a tipping point is a value of a potentially multidimensional threshold variable such that small changes near this value can drive the system to a new stable state at a later point in time (Lamberson & Page, 2012), which could be studied with bifurcation theory (Scheffer *et al*., 2009; Lamberson & Page, 2012; van Nes *et al*., 2016). Thus, tipping points do not necessarily result in an immediate large change; they may simply alter the probability distribution over future states. For instance, if a slight change in the virulence of a disease makes it likely to spread, this qualifies as a tipping point in a system representing a group of animals. If the variable reflecting the system's state, also called the tipping variable, is the same as the threshold variable, it can be referred to as a direct tipping point; otherwise, if they differ, it can be referred to as a contextual tipping point (Lamberson & Page, 2012).

#### (b) Tipping points in welfare studies

Studying the existence of tipping points and related concepts could provide a valuable framework for gaining insight into the mechanisms and temporal properties underlying shifts in an animal or a social system. For example, we may investigate the existence of tipping points that could lead to drastic negative or positive welfare consequences, such as resulting in outbreaks of abnormal behaviours or persistent negative affective state. In the latter case, we may further be interested to study theoretical models to test whether animals in these persistent negative states would require significantly improved environmental conditions to return to their prior state compared to the conditions experienced before passing a tipping point, an idea related to the concept of ‘hysteresis’.

A system is said to exhibit hysteresis if, when reverting the threshold variable in the system that has just passed through a tipping point to the values preceding the tipping point, it fails to restore the system to its previous state (Pruitt *et al*., 2018). For example, in the fictional example from Fig. 4, there exist certain environmental conditions ($T_{1}$ < T < $T_{2}$) where an animal can be either in a positive or negative mood depending on its past states, which indicates that the system exhibits hysteresis. This hysteresis window exists because pigs that have been in a negative mood tend to remain in a negative mood under different environmental conditions ($T_{1}$ < T), whereas pigs that have been in a positive mood tend to remain in a positive mood under different environmental conditions (T < $T_{2}$). Although not all tipping points exhibit hysteresis (Pruitt *et al*., 2018), in practice, when a system shows hysteresis between a welfare indicator and environmental conditions, identifying tipping points could pinpoint moments beyond which addressing the welfare issue may become significantly more expensive than prior to that point.

Identifying tipping points or critical threshold could ultimately be instrumental in developing warning systems aimed at preventing or encouraging changes in animal (social) systems within farm settings. For example, Manning, Chadd & Baines (2007) suggested the potential existence of a direct relationship between changes in daily water consumption and health issues like contact dermatitis and enteritis in broiler chickens. They suggested that future research should identify a threshold level of water consumption at which there will be consequences on litter quality, to help farmers take proactive measures to mitigate future impacts on animal welfare. Studying feedback loops may help to identify tipping points, which could then be integrated into early warning systems signalling the need for intervention at an appropriate moment. However, although certain farmers already collect a range of data including those from behavioural sensors, herd management systems, as well as health information, the potential application of algorithms within farming environments may still be constrained by limitations in data availability (Ledinek *et al*., 2020).

When a system approaches a tipping point, we may want to intervene at one of four facets of the feedback loop underlying the tipping point (*S*, *B*, $\lambda_{B\to S}$, and $\lambda_{S\to B}$) to halt, slow down or accelerate the feedback. For instance, if an abnormal behaviour becomes likely to spread due to a high number of infected animals (e.g. iii in Fig. 3), we could treat animals to reduce the number of infected individuals (*S* variable). Management practices that influence the effects of *S* on *B* or *B* on *S* (i.e. $\lambda_{S\to B}$ or $\lambda_{B\to S}$) could also be used to adjust feedbacks. For example, to interrupt the potential vicious cycle between feather pecking and damaged feathers (Freire *et al*., 1999; McAdie & Keeling, 2000) (ii in Fig. 3), we could adjust the effect of damaged feathers on hens' behaviour ($\lambda_{S\to B}$) by associating feathers with unpalatable substances (Harlander‐Matauschek *et al*., 2008).

An alternative method to interrupt a detrimental positive feedback loop, aside from directly acting on the loop, could involve initiating other feedbacks that have the potential to break a detrimental loop. For example, when trying to disrupt feedbacks between negative moods and pessimistic behaviours, we could generate a negative feedback loop between prediction errors and learning processes (e.g. combining threat cues with a treat may increase prediction errors, thus enhancing learning that some cues should not be perceived as threatening). Similar approaches have been used to attempt to alter negatively biased perceptions of facial expressions in people, and generate more positive responses to ambiguous social information (Suddell *et al*., 2023).

### (3) Eliciting and maintaining positive affective states by generating feedbacks

As a system is maintained through negative feedbacks and can be changed through positive ones, positive feedback loops could provide opportunities to change the state of an animal or a group of animals. Notably, because through positive feedback loops small changes can amplify over time and lead to large changes, even small management interventions could yield substantial and enduring consequences on animals.

In theory, positive feedbacks between a state and a behaviour would lead to an infinite increase in individual differentiation over time (Sih *et al*., 2015). Thus, feedback loops with enduring consequences could theoretically be generated by *simply* triggering a slight increase in one of the two variables. However, in practice, achieving enduring effects may not be as straightforward, since over time we would expect these variables to stabilise due to biological floor and ceiling effects or due to external factors influencing the system, amongst other reasons. Thus, explicitly modelling these feedbacks may enhance our abilities to develop effective management practices that would initiate feedback loops maintained long enough for practical benefits. In practice, continuous‐time dynamic models could estimate how a change in a state predicts a subsequent change in a behaviour after any relevant time lags and the reciprocal effects, thus enabling us to predict whether the desirable outcome would persist, dissipate, or even reverse at some unobserved future time point [see Section IV; e.g. in laying hens (Montalcini *et al*., 2024)].

Ultimately, we could attempt to induce these loops to promote positive affective state. For example, considering that increased oxytocin concentration could induce positive behaviour, which in turn could increase oxytocin concentration, we may try to induce higher basal oxytocin concentration by providing stimuli like brushing and massaging (Chen & Sato, 2017). Similarly, considering that animals in a high positive welfare state may be more likely to engage in environmental challenges that will further enhance their welfare (Franks, Champagne & Higgins, 2013), we could study how to provide cognitive challenges that would initiate relevant feedback loops. However, as increased levels of frustration may decrease performances which, in turn, may further increase frustration, it is important for these challenges to be achievable. Specifically, to account for individual differences in cognition, we could study how to provide farm animals with options to control the level of stimulation (Meagher, 2019), while maintaining animals' motivations over time [e.g. by introducing uncertainty in the acquisition of the reward (Watters, 2009) or by effectively combining different automated learning devices (Langbein, Siebert & Nürnberg, 2009)]. Thus, while cognitive enrichments already exist, we may explore new enrichments by initiating the development process considering potential elements that could trigger and maintain a beneficial positive feedback loop.

## IV. PRACTICAL GUIDE

In addition to studying these feedbacks in conceptual frameworks or theoretical models to understand mechanisms underpinning certain welfare issues better, we may also explore state–behaviour feedback loops in experimental studies, by estimating the influence of a state (*S*) on a behaviour (*B*) ($\lambda_{S\to B}$) and of *B* on *S* ($\lambda_{B\to S}$) within a single model. We will here briefly discuss data requirements and potential methods to achieve this. Note that these methods may in theory be applied to any two variables, not exclusively to a state and a behaviour.

### (1) Data requirements

Such an analysis requires longitudinal data on both *S* and *B* for at least one individual. The required number of observations per individual depends on several factors, including the effect size of the temporal effects ($\lambda_{S\to B}$ and $\lambda_{B\to S}$), the number of individuals, and the chosen statistical approach, among others. Therefore, it is not possible to provide a general indication of the required sample size. While it is encouraged to have a large data set for such analysis, the approach discussed below – based on hierarchical Bayesian continuous time dynamic models (Driver & Voelkle, 2018a) – also works with data from a single individual with multiple observations. Moreover, Bayesian estimations are generally well‐suited for modelling data with small sample sizes, provided that appropriate prior distributions are used (McNeish, 2016).

### (2) Potential statistical approaches

As suggested by Sih *et al*. (2015), feedback loops could be explored using a ‘reaction norm’ approach (Dingemanse *et al*., 2010) where the environmental gradient is represented by a ‘time’ variable. First, we could fit two mixed‐effect models with random intercepts and slopes (one model for the state and one for the behaviour). Correlations between these random effects would indicate whether state and behaviour showed patterns of ‘fanning‐in’ (Fig. 1B,D; indicated by negative correlations) or ‘fanning‐out’ (Fig. 1C,E; indicated by positive correlations). To differentiate positive loops with temporal effects of positive signs ($\lambda_{S\to B}>0$ and $\lambda_{B\to S}>0$, Fig. 1C) from those with temporal effects of negative signs ($\lambda_{S\to B}<0$ and $\lambda_{B\to S}<0$, Fig. 1E), we could also estimate within‐individual correlations between the state and behaviour variables and expect positive and negative correlations for effects with positive and negative signs, respectively. Sih *et al*. (2015) suggested then also to fit a bivariate mixed‐effect model with random intercepts and slopes, using state and behaviour as response variables. In the presence of positive or negative feedback loops, the slopes of the reaction norms for state and behaviour should be correlated.

While the reaction norm approach would estimate how a state and a behaviour change within individuals over time, it will not estimate the effect of state on behaviour and *vice versa*. These temporal effects ($\lambda_{S\to B}$ and $\lambda_{B\to S}$) could be estimated using structural equation modelling, as also suggested by Sih *et al*. (2015), using discrete‐ or continuous‐time models (e.g. see Fig. 1A or Driver, Oud & Voelkle, 2017). Continuous‐time models offer many advantages, such as accommodating unequally spaced data (Voelkle *et al*., 2012; Driver & Voelkle, 2018b), better representing continually evolving processes (Oud, 2002; Oud & Delsing, 2010), and enabling the comparison of temporal effects across studies with different time intervals between measurements (van Montfort, Oud & Voelkle, 2018). Indeed, by providing temporal effects estimates for different time intervals between observations, continuous‐time models can shed light on whether effects are maintained, dissipated, or eventually reverse with time, and thus may aid in explaining results that may seem contradictory. Also, by estimating how the system changes as a function of current state rather than past, these models separate the indirect effects from the direct effects driving the changes. To the extent that the model is a fair representation of reality, this allows for inference of causal effects (Moggia *et al*., 2023). Yet, despite these advantages, the use of continuous‐time dynamic models in the behavioural sciences remains limited (van Montfort *et al*., 2018).

Recent models developed by Driver & Voelkle (2018a), called hierarchical Bayesian continuous‐time dynamic models and implemented in the *ctsem* R package (Driver *et al*., 2017), may be relevant to study feedback loops between a state and a behaviour. These models are based on coupled stochastic differential equations and estimate latent variables from (multiple) observed variable(s), typically called indicators. Latent variables generally refer to variables that cannot be directly measured with perfect accuracy, and are instead inferred from observable measurements. These are widely used in psychology to study human personality, intelligence, mood, and motivation. To represent better the subjectivity of non‐human animals' affective states, we could, for example, use these and other techniques to estimate a latent variable from multiple observed variables [e.g. heart rate variability, facial expression, and vocalisations (Neethirajan, Reimert & Kemp, 2021; Coleman & Neethirajan, 2022)]. By allowing for individual variation in the temporal effects ($\lambda_{S\to B}$ and $\lambda_{B\to S}$), we can also estimate within‐individual feedbacks. We can also account for moderation effects on $\lambda_{S\to B}$ and/or $\lambda_{B\to S}$.

In the online Supporting Information, Appendix S1, we provide annotated R code that demonstrates the use of hierarchical Bayesian continuous‐time dynamic modelling to estimate how a change in a behaviour predicts changes in a state, and *vice versa*, at both the population‐level (Section 1) and individual‐level (Section 2). If the behaviour and state variables involved in the dynamical system vary systematically as a function of time (or another known variable), we should either detrend the two variables prior to fitting the model or model the trend and assess the dynamic between their respective fluctuations around their trend. The latter approach makes best use of available information and avoids conditioning inferences on a single trend (when this may be highly uncertain), although it is also more complex. We implement this approach in Section 3 of the R code, which is further detailed in Driver & Tomasik (2022).

## V. CONCLUSIONS

(1) Our current understanding of animal behaviour is mainly based on static ‘snapshots’ rather than ongoing feedback loops that capture behaviour in response to continuously changing individual, social, and environmental states. Thus, many feedback loops have been proposed in previous literature but await empirical testing.
(2) We find evidence in support of state–behaviour feedback loops with consequences for farm animal welfare in various livestock species, including chickens, pigs, horses, mink, and rabbits, alongside more general examples applicable to various species. Some of these examples could also be relevant for the welfare of other captive animals.
(3) We highlight various state variables relevant for examining these feedback loops, including characteristics related to an individual's affective state, health, learning state, hormonal state, and motivational state, as well as those related to the individual's social environment.
(4) Potential consequences of these loops for animal welfare are diverse and include prolonged positive or negative affective states, development of behavioural competence, prolonged positive or negative social experiences, increased physical impairments, increased expression of abnormal and damaging behaviour, and increased parasite load.
(5) Most of the examples discussed in this review are detrimental rather than beneficial to animal welfare, which may be partially due to the prevailing focus on negative rather than positive state in farm animal literature, further underscoring the need to study positive states (Lawrence, Vigors & Sandøe, 2019).
(6) This paper focus on state–behaviour feedbacks, but extensions could consider feedbacks between two behaviours or two states. For example, given that stress is recognised as a risk factor for certain health issues, and that in turn health issues can exacerbate stress levels, we may explore detrimental loops between stress and health issues.
(7) Through this work, we hope to encourage use of dynamic models that integrate longitudinal data on animals' behaviour and state to enable exploration of their dynamics and ultimately foster new perspectives for improving farm animal welfare.

## Supporting information


**Appendix S1.** State–behaviour dynamic – a practical guide.
