# Supplementary material for: Relevance of state–behaviour feedbacks for animal welfare
Source: Biol Rev Camb Philos Soc. 2025 Mar 24;100(4):1615–34. doi: 10.1111/brv.70016 (PMC12227790; doi:10.1111/brv.70016)
Supplement: Supplementary file 1 — Appendix S1. State–behaviour dynamic – a practical guide. [file BRV-100-1615-s001.html]

State–behaviour dynamic – A practical guide


# State–behaviour dynamic – A practical guide

#### 2025-03-11

```
library(ggplot2)
library(ctsem)
```

# 1. Dynamics between a state (*S*) and a behaviour (*B*) at the population level

### 1.1 Generating Data

We here generate data for 40 individuals, generating observations of
state and behaviour once per day, over a period of 40 days. Individuals
differ in their initial state and behaviour levels. We assume a positive
correlation between the random fluctuations of the state and the
behaviour, implying that events occurring in an animal’s life may
generate fluctuations in the state and the behaviour in the same
direction. Importantly, we include continuous-time temporal
cross-effects, which captures how the state and the behaviour are
influenced by each other.

This model captures the idea that both state and behaviour could be
subject to both predictable trends (e.g. due to an aging effect;
captured by their rate of change represented in the continuous-time
temporal auto-effects called “A”) as well as random fluctuations
(captured by the coefficient called “Gcross” from the system noise),
reflecting some of the complexity of real-world state (or behaviour)
dynamics.

Temporal effects are contained in a drift matrix, where the cross-
(off-diagonal) and auto- (diagonal) effects can be interpreted
similarly: a positive (negative) effect indicates that higher values of
the causal process led to rises (drops) in the caused process. Assuming
that there are indeed fluctuations around the baseline trend, the
continuous-time auto-effects are expected to be negative, with more
negative effects indicating a stronger force pushing the deviations back
to baseline – this implies that changes away from the baseline dissipate
faster and the process more frequently crosses its baseline.

Relevant points when working with real-world data:

- Missing data for state and or behaviour variables are not a problem,
  so long as (a) there is a sufficient number of observations to estimate
  the processes, and (b) the cause of the missingness is unrelated to the
  outcome (standard missing at random assumption).
- if alignment of trends across time is relevant, as it may be when
  the time variable is age: for individuals that do not have observations
  at time=0, one should create an empty observation row for these
  individuals at time=0 and set observed variables to NA
- this approach works well with varying time intervals within and
  between individuals.

```
set.seed(123) # Set random seed for reproducibility
# Generate data for 40 individuals (here also called "Subject") with individual differences
NSubjects <- 40
times <- seq(from=0, to=40, by=1) #generate sequence of time points when subjects are measured
Nobs <- length(times) #number of observations per subject
initialState <- rnorm(NSubjects, mean = 5, sd = 2)
initialBehaviour <- rnorm(NSubjects, mean = 5, sd = 2)
A <- -0.3 #continuous time auto-effects (i.e. state dependence over its past values and behaviour dependence over its past values )
AcrossSB <- 0.15 #continuous time cross-effect: S-->B
AcrossBS <- 0.1  #continuous time cross-effect: B-->S
CS <- 1 #continuous intercept for S
CB <- 1.5 #continuous intercept for B
G <- 0.2 #unique system noise coefficient
Gcross <- 0.1 #common system noise coefficient

#create empty data.frame to fill step by step
data <- data.frame(Subject= rep(NA,NSubjects*Nobs), 
  Time = rep(NA,NSubjects*Nobs), 
  State = rep(NA,NSubjects*Nobs),
  Behaviour = rep(NA,NSubjects*Nobs)) #now with affect for two individuals

Nsteps <- 100 #number of steps in time to compute between each observation (increased computational accuracy)

rowCounter <- 0 #initialize row counter, to track which row of the data.frame we are on
for(subjecti in 1:NSubjects){
  for(obsi in 1:Nobs){ #for each observation of a subject
    rowCounter <- rowCounter + 1
    if(obsi==1){
      State <- initialState[subjecti] #if first time point, set to initial variable
      Behaviour <- initialBehaviour[subjecti]
    }
    if(obsi>1){ #else compute new value by taking a sequence of small steps in time
      for(stepi in 1:Nsteps){ #take Nsteps in time between each observation
        dState <- A*State + AcrossBS * Behaviour + CS #compute deterministic slope of affect at earlier time point
        dBehaviour <- A*Behaviour + AcrossSB * State + CB
        
        systemNoiseS <- rnorm(n=1, mean=0, sd=sqrt(1/Nsteps)) #unique noise for individual 1
        systemNoiseB <- rnorm(n=1, mean=0, sd=sqrt(1/Nsteps)) #unique noise for individual 2
        systemNoiseCross <- rnorm(n=1, mean=0, sd=sqrt(1/Nsteps)) #common noise for both individuals
        
        State <- State + dState * 1/Nsteps + #update state using slope and time step
          G * systemNoiseS + Gcross * systemNoiseCross #and add unique and common system noise
        Behaviour <- Behaviour + dBehaviour * 1/Nsteps + #update variable using slope and time step
          G * systemNoiseB + Gcross * systemNoiseCross #and add unique and common system noise
      }
    }
    data$State[rowCounter] <- State #input state data
    data$Behaviour[rowCounter] <- Behaviour #input behaviour data
    data$Time[rowCounter] <- times[obsi] #input time data
    data$Subject[rowCounter] <- subjecti #input subject data
  }
}

data$State <- data$State + rnorm(n=nrow(data), mean = 0, sd = .05) #add measurement error
data$Behaviour <- data$Behaviour + rnorm(n=nrow(data), mean = 0, sd = .05) #add measurement error

head(data, 3)
```

```
##   Subject Time    State Behaviour
## 1       1    0 3.866772  3.654283
## 2       1    1 4.231087  4.561671
## 3       1    2 4.482774  4.999394
```

In theory, positive feedbacks between a state and a behaviour would
lead to an infinite increase in both variables over time. However, in
practice, we would expect these variables to stabilise over time due to
biological floor and ceiling effects or due to external factors
influencing the system, among others, and this concept is represented by
the negative auto-effects. Each of the two figures below shows
instantiations of one random individual’s possible measurements.

```
#state and behaviour over time for the first individual
ggplot(data[data$Subject == 1, ], aes(x = Time)) +
  geom_line(aes(y = State, color = "blue")) +
  geom_line(aes(y = Behaviour, color = "red")) +
  geom_point(aes(y = State), color = "blue") +
  geom_point(aes(y = Behaviour), color = "red") +
  theme_bw() +
  labs(title = "State (blue) and Behaviour (red) over Time", x = "Time", y = "Affect") +
  scale_color_manual(values = c("blue", "red")) +
  theme(legend.position = "none")
```

```
#state and behaviour over time for the second individual
ggplot(data[data$Subject == 2, ], aes(x = Time)) +
  geom_line(aes(y = State, color = "blue")) +
  geom_line(aes(y = Behaviour, color = "red")) +
  geom_point(aes(y = State), color = "blue") +
  geom_point(aes(y = Behaviour), color = "red") +
  theme_bw() +
  labs(title = "State (blue) and Behaviour (red) over Time", x = "Time", y = "Affect") +
  scale_color_manual(values = c("blue", "red")) +
  theme(legend.position = "none")
```

### 1.2 ctsem Model Specification

We now specify the continuous-time structural equation model (ctsem)
to estimate the dynamics between the state and the behaviour. The model
includes continuous-time auto-effects (state dependence over its past
values and behaviour dependence over its past values), a continuous-time
cross-effect (from state to behaviour and vice versa), and system noise
for the state and the behaviour. The model also includes a continuous
intercept, which represents the long-term equilibrium level of the state
and the behaviour. In addition, we estimate the initial state and
behaviour levels for each individual, as well as the standard deviation
of the residual (measurement error) for the state and the behaviour.
Names of the variables are defined via the manifestNames argument, and
the latent process names are defined via the latentNames argument. The
time variable is defined via the time argument, and the subject variable
is defined via the id argument. The type argument specifies the type of
model to be used (stanct for continuous-time models). The LAMBDA matrix
specifies the relationship between the latent processes and the observed
variables.

```
ct_model <- ctModel( #define the ctsem model
  manifestNames = c("State",'Behaviour'), #names of observed variables in dataset
  latentNames = c("State",'Behaviour'), #names of latent processes
  time = 'Time', #name of time column in dataset
  id = 'Subject', #name of subject column in dataset
  type='stanct', #use continuous time / differential equation model (standt for discrete-time / regression model)
  MANIFESTVAR = c('residualSD1',0,
                  0, 'residualSD2'), #sd of the residual / measurement error
  LAMBDA = diag(1,2), #relating latent process to observed variables
  MANIFESTMEANS=0, #no measurement intercept / offset needed (1 observed variable relates directly to latent)
  CINT=c('timeCoefficient1||FALSE','timeCoefficient2||FALSE'), #continuous intercept with *no* random effects
  T0MEANS=c('initialState||TRUE','initialBehaviour||TRUE'), #initial affect with random effects
  DRIFT = c('autoEffectS', 'crossEffectBS', #state dependence  over its past values and cross-effect from behaviour to state
            'crossEffectSB','autoEffectB' ), #cross-effect from state to behaviour and behaviour dependence over its past values
  DIFFUSION = c('systemNoiseS', 0, #system noise for state variable, 0 in upper triangle (because correlation only needs 1 par)
                'systemNoiseCross', 'systemNoiseB')) #correlation in system noise, and sd for behaviour variable
```

```
ctModelLatex(ct_model) #generate LaTeX representation of the model
```

### 1.3 Fit and Summarise ctsem Model

We now fit the model to our data. By default, this uses maximum
likelihood. If you want to use priors for a Bayesian approach, this can
be requested by setting priors=TRUE. Please refer to the ctsem
documentation by running `ctDocs()` for more details.

```
ct_fit <- ctStanFit(datalong = data, ctstanmodel = ct_model) #fit the model to our data
```

```
## [1] 450.882
## attr(,"gradient")
##  [1]  2.449816e-01 -1.392534e-02  9.705309e+02  2.094760e+03 -7.507650e+02
##  [6] -4.978559e+02 -5.030484e+02  4.261005e+01  3.090718e+01 -1.757907e+02
## [11]  4.678148e-03  2.734113e+03 -1.273745e+03 -2.731527e-01  1.082822e-03
## [16] -2.438071e-02
```

```
summary(ct_fit, parmatrices=FALSE) #print summary of the fit, some output disabled
```

```
## $residCovStd
##           State Behaviour
## State     0.336     0.034
## Behaviour 0.034     0.168
## 
## $resiCovStdNote
## [1] "Standardised covariance of residuals"
## 
## $rawpopcorr
##                                  mean     sd    2.5%    50%  97.5%      z
## initialBehaviour__initialState 0.0528 0.1561 -0.2559 0.0567 0.3619 0.3383
## 
## $popsd
##                    mean     sd   2.5%    50%  97.5%
## initialState     1.8002 0.2003 1.4222 1.7930 2.2108
## initialBehaviour 1.9139 0.2174 1.5216 1.9064 2.3737
## 
## $popmeans
##                     mean     sd    2.5%     50%   97.5%
## initialState      5.1025 0.2836  4.5233  5.1213  5.6334
## initialBehaviour  4.9663 0.3030  4.3700  4.9676  5.5699
## autoEffectS      -0.3218 0.0144 -0.3507 -0.3213 -0.2951
## crossEffectBS     0.1014 0.0072  0.0871  0.1016  0.1155
## crossEffectSB     0.1241 0.0136  0.0994  0.1236  0.1522
## autoEffectB      -0.3022 0.0094 -0.3203 -0.3020 -0.2842
## systemNoiseS      0.2316 0.0017  0.2283  0.2316  0.2349
## systemNoiseCross  0.1112 0.0134  0.0851  0.1116  0.1377
## systemNoiseB      0.2330 0.0014  0.2303  0.2331  0.2358
## residualSD1       0.0000 0.0000  0.0000  0.0000  0.0000
## residualSD2       0.0000 0.0000  0.0000  0.0000  0.0000
## timeCoefficient1  1.1173 0.0418  1.0396  1.1173  1.1987
## timeCoefficient2  1.6633 0.0662  1.5305  1.6635  1.7913
## 
## $popNote
## [1] "popmeans are reported as specified in ctModel -- covariance related matrices are in sd / unconstrained correlation form -- see $parmatrices for simpler interpretations!"
## 
## $loglik
## [1] 475.5449
## 
## $npars
## [1] 16
## 
## $aic
## [1] -919.0897
## 
## $logposterior
## [1] 475.5449
## 
## $parmatNote
## [1] "For additional summary matrices, use argument: parmatrices = TRUE"
```

In the summary output, we can see the estimated parameters, including
the continuous-time auto-effects (autoEffectS and autoEffectB), the
continuous-time cross-effects (crossEffectBS and crossEffectSB), the
system noise (systemNoiseS and systemNoiseB), and the system noise
correlation (systemNoiseCross). The estimated parameters are presented
in the `$popmeans` section which shows the estimated
population means and uncertainty of the parameter estimates. The
`$popsd` section shows the estimated population standard
deviations of any parameters for which random effects were estimated.
The `$rawpopcorr` section shows the estimated correlations
between the parameters.

### 1.4 Visualise Predictions

We can visualise predictions from this model, including multivariate
dynamics, state dependence, behaviour dependence, and random
fluctuation. The model represents the complex interplay between the
individuals’ state and behaviour, and the uncertainty in the predictions
reflects two sources. The first is that there are inherently
unpredictable fluctuations in both variables due to factors we have not
observed or explicitly modelled, so the deterministic portion of the
model is inevitably imperfect. The second source of uncertainty is that
each measurement of state or behaviour is likely an imperfect indicator
of the underlying state and behaviour, so we can’t be sure of the true
state and behaviour of the system even at the moment where we measure
it. Here we’ll look at predictions based on the estimated model
parameters and every 5th data point for our 3rd animal (subject 3).

```
ctKalman(fit = ct_fit, plot=TRUE, subjects = 3, kalmanvec=c('y','yprior'), removeObs = 5)
```

### 1.5 Visualise Dynamics

To understand more easily the dynamics implied by the estimated model
parameters, we can compute the discrete-time auto and cross regression
coefficients, which tell us how an individual’s state can be predicted
by its earlier values, and by its behaviour; and how an individual’s
behaviour can be predicted by its earlier values, and by its state.
Specifically, while continuous-time temporal effects describe how the
process is changing at the moment, discrete-time cross-regressions for a
time interval of *k* represent the effect of one variable at
earlier time *t-k* on another variable at later time *t*.
We compute these across a range of times and then plot them. This
visualisation can also be interpreted as the expected change in the
system, conditional on observing a change of magnitude 1 in the causal
(column) variable – this is otherwise known as an impulse response
function.

```
ctStanDiscretePars(ct_fit,plot=T) #plot dynamics
```

An important variation on such a plot is to consider also the
estimated correlation in the system noise, which tells us how much the
fluctuations in the state and the behaviour are related, and how this
plays out across time. This essentially means that if random-effects on
state and behaviour (i.e. system noise) are positively correlated, when
an individual experiences a random fluctuation in state, its behaviour
is also likely to experience a fluctuation in the same direction. Thus,
showing predictions conditional on only one variable changing may not be
so informative, particularly when the system noise correlation is
strongly positive or negative. In such cases, the visualised
cross-effects may be misleading, as they represent the expected change
in the system when only one of the variables experience a change, but in
reality, the other variable is also likely to experience a change at the
same time. For more on the combined interpretation of system noise and
dynamics, see https://doi.org/10.31234/osf.io/y3e9d.

```
ctStanDiscretePars(ct_fit,plot=T, observational=TRUE) #plot dynamics
```

From this plot we see that indeed, the individual’s state is expected
to increase when we observe a positive change in its behaviour, and vice
versa. Here, we can be sure that there is a causal effect because we
generated the data with such an effect, but in a real-world scenario, we
would need to consider the possibility of confounding variables that
could explain the observed relationship. The system noise correlation
can account for confounders that change very quickly (compared to the
speed of change of affect), but is unlikely to sufficiently account for
confounders that change at a similar speed to affect. Slowly changing
confounders are not accounted for in this model, but individual
differences in the continuous intercept (setting long-term equilibrium
levels) could be easily turned back on to account for some such
effects.

# 2. Within-individual *S*–*B* dynamics

Until now we assessed the dynamics between a state and a behaviour at
the population-level. To study within-individual feedback loops, we here
allow individual variation in the dynamics (i.e. in the continuous-time
temporal cross-effects, represented in the drift matrix (off-diagonal
entries)).

### 2.1 Generating Data

We generate data for 40 individuals, generating observations of state
and behaviour once per day over a period of 40 days. Individuals differ
in their initial state and behaviour levels as before, but now also in
the continuous-time temporal cross-effects. We assume a positive
correlation between the random fluctuations of the state and the
behaviour, implying that events occurring in an animal’s life may
generate fluctuations in the state and the behaviour in the same
direction.

```
set.seed(123) # Set random seed for reproducibility
# Generate data for 40 individuals (here also called "Subject") with individual differences
NSubjects <- 40
times <- seq(from=0, to=40, by=1) #generate sequence of time points when subjects are measured
Nobs <- length(times) #number of observations per subject
initialState <- rnorm(NSubjects, mean = 5, sd = 2)
initialBehaviour <- rnorm(NSubjects, mean = 5, sd = 2)

A <- -0.3 #continuous time auto-effects 
AcrossSB <- rnorm(NSubjects, mean = 0.15, sd = 0.05) #continuous time cross-effect (S-->B), now with individual differences 
AcrossBS <- rnorm(NSubjects, mean = 0.1, sd = 0.05)  #continuous time cross-effect (B-->S), now with individual differences 
CS <- 1 #continuous intercept for S
CB <- 1.5 #continuous intercept for B
G <- 0.2 #unique system noise coefficient
Gcross <- 0.1 #common system noise coefficient

#create empty data.frame to fill step by step
data <- data.frame(Subject= rep(NA,NSubjects*Nobs), 
  Time = rep(NA,NSubjects*Nobs), 
  State = rep(NA,NSubjects*Nobs),
  Behaviour = rep(NA,NSubjects*Nobs)) #now with affect for two individuals

Nsteps <- 100 #number of steps in time to compute between each observation (increased computational accuracy)

rowCounter <- 0 #initialize row counter, to track which row of the data.frame we are on
for(subjecti in 1:NSubjects){
  for(obsi in 1:Nobs){ #for each observation of a subject
    rowCounter <- rowCounter + 1
    if(obsi==1){
      State <- initialState[subjecti] #if first time point, set to initial variable
      Behaviour <- initialBehaviour[subjecti]
    }
    if(obsi>1){ #else compute new value by taking a sequence of small steps in time
      for(stepi in 1:Nsteps){ #take Nsteps in time between each observation
        dState <- A*State +  AcrossBS[subjecti] * Behaviour + CS #compute deterministic slope of affect at earlier time point
        dBehaviour <- A*Behaviour +  AcrossSB[subjecti] * State + CB
        
        systemNoiseS <- rnorm(n=1, mean=0, sd=sqrt(1/Nsteps)) #unique noise for individual 1
        systemNoiseB <- rnorm(n=1, mean=0, sd=sqrt(1/Nsteps)) #unique noise for individual 2
        systemNoiseCross <- rnorm(n=1, mean=0, sd=sqrt(1/Nsteps)) #common noise for both individuals
        
        State <- State + dState * 1/Nsteps + #update state using slope and time step
          G * systemNoiseS + Gcross * systemNoiseCross #and add unique and common system noise
        Behaviour <- Behaviour + dBehaviour * 1/Nsteps + #update variable using slope and time step
          G * systemNoiseB + Gcross * systemNoiseCross #and add unique and common system noise
      }
    }
    data$State[rowCounter] <- State #input state data
    data$Behaviour[rowCounter] <- Behaviour #input behaviour data
    data$Time[rowCounter] <- times[obsi] #input time data
    data$Subject[rowCounter] <- subjecti #input subject data
  }
}

data$State <- data$State + rnorm(n=nrow(data), mean = 0, sd = .05) #add measurement error
data$Behaviour <- data$Behaviour + rnorm(n=nrow(data), mean = 0, sd = .05) #add measurement error

head(data, 3)
```

```
##   Subject Time    State Behaviour
## 1       1    0 3.903871  3.603724
## 2       1    1 4.057643  4.764585
## 3       1    2 4.236156  5.430934
```

### 2.2 ctsem Model Specification

In the following, we adapt the earlier model specification code to
allow for individual differences in the continuous-time temporal
cross-effects. We also set a tight prior for the individual variation in
the drift effects, as we expect the individual differences to be
small.


```
ct_model <- ctModel( #define the ctsem model
  manifestNames = c("State",'Behaviour'), #names of observed variables in dataset
  latentNames = c("State",'Behaviour'), #names of latent processes
  time = 'Time', #name of time column in dataset
  id = 'Subject', #name of subject column in dataset
  type='stanct', #use continuous time / differential equation model (standt for discrete-time / regression model)
  MANIFESTVAR = c('residualSD1',0,
    0, 'residualSD2'), #sd of the residual / measurement error
  LAMBDA = diag(1,2), #relating latent process to observed variables
  MANIFESTMEANS=0, #no measurement intercept / offset needed (1 observed variable relates directly to latent)
  CINT=c('timeCoefficient1||FALSE','timeCoefficient2||FALSE'), #continuous intercept with *no* random effects
  T0MEANS=c('initialState||TRUE','initialBehaviour||TRUE'), #initial affect with random effects
  DRIFT = c('autoEffectS', 'crossEffectBS||TRUE|.05', #temporal effects now specify some random effects, with a prior sd of .05
    'crossEffectSB||TRUE|.05','autoEffectB' ), 
  DIFFUSION = c('systemNoiseS', 0, #system noise for state variable, 0 in upper triangle (because correlation only needs 1 par)
    'systemNoiseCross', 'systemNoiseB')) #correlation in system noise, and sd for behaviour variable
```

### 2.3 Fit and Summarise ctsem Model

We now fit the model to our data. By default, this uses maximum
likelihood. If you want to use priors for a Bayesian approach, this can
be requested by setting priors=TRUE. Please refer to the ctsem
documentation by running `ctDocs()` for more details.

```
ct_fit <- ctStanFit(datalong = data, ctstanmodel = ct_model) #fit the model to our data
```

```
## Error in sgd(init, fitfunc = target, parsets = parsets, nsubsets = 1,  : 
##   NA in parameter proposal!
```

```
summary(ct_fit, parmatrices=FALSE) #print summary of the fit, some output disabled
```

```
## $residCovStd
##           State Behaviour
## State     0.047     0.007
## Behaviour 0.007     0.066
## 
## $resiCovStdNote
## [1] "Standardised covariance of residuals"
## 
## $rawpopcorr
##                                    mean     sd    2.5%     50%  97.5%       z
## initialBehaviour__initialState   0.0523 0.1514 -0.2599  0.0479 0.3512  0.3451
## crossEffectBS__initialState      0.0273 0.1547 -0.2769  0.0305 0.3274  0.1767
## crossEffectSB__initialState      0.0053 0.1491 -0.2946  0.0101 0.2761  0.0352
## crossEffectBS__initialBehaviour  0.2076 0.1420 -0.0658  0.2121 0.4835  1.4621
## crossEffectSB__initialBehaviour -0.0765 0.1529 -0.3582 -0.0833 0.2348 -0.5004
## crossEffectSB__crossEffectBS     0.1182 0.1536 -0.1904  0.1161 0.4070  0.7697
## 
## $popsd
##                    mean     sd   2.5%    50%  97.5%
## initialState     1.7910 0.2046 1.4317 1.7739 2.2194
## initialBehaviour 1.9050 0.2168 1.5254 1.8993 2.3781
## crossEffectBS    0.0537 0.0067 0.0410 0.0534 0.0679
## crossEffectSB    0.0447 0.0054 0.0346 0.0446 0.0556
## 
## $popmeans
##                     mean     sd    2.5%     50%   97.5%
## initialState      5.0987 0.2779  4.5856  5.0961  5.6445
## initialBehaviour  4.9729 0.3007  4.3543  4.9664  5.5807
## autoEffectS      -0.3129 0.0143 -0.3395 -0.3130 -0.2856
## autoEffectB      -0.3036 0.0093 -0.3214 -0.3036 -0.2852
## systemNoiseS      0.2156 0.0086  0.1994  0.2157  0.2332
## systemNoiseCross  0.0939 0.0164  0.0607  0.0943  0.1279
## systemNoiseB      0.2147 0.0079  0.2003  0.2146  0.2309
## residualSD1       0.0668 0.0122  0.0461  0.0660  0.0940
## residualSD2       0.0612 0.0123  0.0400  0.0599  0.0883
## timeCoefficient1  1.1048 0.0704  0.9753  1.1026  1.2488
## timeCoefficient2  1.6428 0.0689  1.5101  1.6444  1.7678
## crossEffectBS     0.0901 0.0140  0.0626  0.0898  0.1178
## crossEffectSB     0.1284 0.0150  0.0997  0.1281  0.1586
## 
## $popNote
## [1] "popmeans are reported as specified in ctModel -- covariance related matrices are in sd / unconstrained correlation form -- see $parmatrices for simpler interpretations!"
## 
## $loglik
## [1] 269.8655
## 
## $npars
## [1] 23
## 
## $aic
## [1] -493.7309
## 
## $logposterior
## [1] 269.8655
## 
## $parmatNote
## [1] "For additional summary matrices, use argument: parmatrices = TRUE"
```

We now can see in the `$popsd` that in addition to the
initialState and initialBehaviour, we also have the cross-effects, and
in the `$rawpopcorr` the correlation between the
cross-effects and initial states.

### 2.4 Visualise Individual Dynamics

We can look at predictions based on the estimated model parameters as
in 1.4. Here, we show how to visualise the individual differences in the
dynamics that result from differences in the cross-effects, for two
different individuals:

```
ctStanDiscretePars(ct_fit,plot=T, subjects=c(2,4), observational=TRUE)+facet_wrap(~Subject)
```

### 2.5 Individual Difference in Temporal Effects

We can extract estimates of individual specific parameters from the
model and visualise them. Here we show the individual differences in the
cross-effects, which represent the effect of behaviour on state and vice
versa, and the corresponding boxplot.

```
library(data.table)
library(ggplot2)
subjectPars <- ctStanSubjectPars(ct_fit)[1,,] #extra point estimate of individual specific parameters
subjectPars <- melt(data.table(subject=rownames(subjectPars), subjectPars), id.vars = "subject") #reshape data for ggplot
ggplot(subjectPars, aes(y = value,colour=variable)) +
  geom_boxplot()+
  geom_point(mapping=aes(x=0),position=position_jitter(width=.05))+
  theme_bw() +
  facet_wrap(~variable, scales = "free") +
  labs(title = "Individual differences in model parameters")+
  xlab('')
```

# 3. *S*–*B* dynamics with temporal trend in *S* and *B*

If the variables (*S*, *B*) involved in the dynamical
system vary systematically as a function of time (or another known
variable), we should either detrend the *S* and *B*
variables prior to fitting the model or model the trend and assess the
dynamic between their respective fluctuation around their trend. The
latter approach makes best use of available information and avoids
conditioning inferences on a single trend (when this may be highly
uncertain), although it is also more complex.

To model separate trends around which we would estimate the dynamics,
we need to extend the number of latent processes in the model and have
each of them accounting for one trend process (without system noise),
while the original processes in the model account for the fluctuations
and their dynamics. We provide here an R code illustrating this, which
is described in detail in Driver & Tomasik (2022). For simplicity,
we’ve gone back to a single cross-effect parameter in the population,
without individual differences.

### 3.1 Generating Data

```
set.seed(123) # Set random seed for reproducibility
# Generate data for 40 individuals (here also called "Subject") with individual differences
NSubjects <- 40
times <- seq(from=0, to=40, by=1) #generate sequence of time points when subjects are measured
Nobs <- length(times) #number of observations per subject
initialState <- rnorm(NSubjects, mean = 5, sd = 2)
initialBehaviour <- rnorm(NSubjects, mean = 5, sd = 2)

A <- -0.3 #continuous time auto-effects (i.e. state dependence over its past values and behaviour dependence over its past values )
AcrossSB <- 0.15 #continuous time cross-effect: S-->B
AcrossBS <- 0.1  #continuous time cross-effect: B-->S
CS <- 1 #continuous intercept for S
CB <- 1.5 #continuous intercept for B
G <- 0.2 #unique system noise coefficient
Gcross <- 0.1 #common system noise coefficient

StateTrend <- rnorm(NSubjects, mean = -.8, sd = 0.1) #individual trend in state
BehaviourTrend <- StateTrend * .3 + rnorm(NSubjects, mean = -.4, sd = 0.1) #trend in behaviour, correlated with trend in state

#create empty data.frame to fill step by step
data <- data.frame(Subject= rep(NA,NSubjects*Nobs), 
  Time = rep(NA,NSubjects*Nobs), 
  State = rep(NA,NSubjects*Nobs),
  Behaviour = rep(NA,NSubjects*Nobs)) #now with affect for two individuals

Nsteps <- 100 #number of steps in time to compute between each observation (increased computational accuracy)

rowCounter <- 0 #initialize row counter, to track which row of the data.frame we are on
for(subjecti in 1:NSubjects){
  for(obsi in 1:Nobs){ #for each observation of a subject
    rowCounter <- rowCounter + 1
    if(obsi==1){
      State <- initialState[subjecti] #if first time point, set to initial variable
      Behaviour <- initialBehaviour[subjecti]
    }
    if(obsi>1){ #else compute new value by taking a sequence of small steps in time
      for(stepi in 1:Nsteps){ #take Nsteps in time between each observation
        dState <- A*State +  AcrossBS * Behaviour + CS  #compute deterministic slope of affect at earlier time point
        dBehaviour <- A*Behaviour +  AcrossSB * State + CB
        
        systemNoiseS <- rnorm(n=1, mean=0, sd=sqrt(1/Nsteps)) #unique noise for individual 1
        systemNoiseB <- rnorm(n=1, mean=0, sd=sqrt(1/Nsteps)) #unique noise for individual 2
        systemNoiseCross <- rnorm(n=1, mean=0, sd=sqrt(1/Nsteps)) #common noise for both individuals
        
        State <- State + dState * 1/Nsteps + #update state using slope and time step
          G * systemNoiseS + Gcross * systemNoiseCross + #and add unique and common system noise
          StateTrend[subjecti] * 1/Nsteps #add trend
        Behaviour <- Behaviour + dBehaviour * 1/Nsteps + #update variable using slope and time step
          G * systemNoiseB + Gcross * systemNoiseCross + #and add unique and common system noise
          BehaviourTrend[subjecti] * 1/Nsteps #add trend
      }
    }
    data$State[rowCounter] <- State #input state data
    data$Behaviour[rowCounter] <- Behaviour #input behaviour data
    data$Time[rowCounter] <- times[obsi] #input time data
    data$Subject[rowCounter] <- subjecti #input subject data
  }
}

data$State <- data$State + rnorm(n=nrow(data), mean = 0, sd = .05) #add measurement error
data$Behaviour <- data$Behaviour + rnorm(n=nrow(data), mean = 0, sd = .05) #add measurement error

head(data, 3)
```

```
##   Subject Time    State Behaviour
## 1       1    0 3.903871  3.603724
## 2       1    1 3.317272  4.168650
## 3       1    2 2.894562  4.307331
```

### 3.2 ctsem Model Specification

Note here that we have added two new latent processes, StateTrend and
BehaviourTrend, to account for the systematic variation in the state and
behaviour over time. We also added the corresponding trend parameters in
the drift matrix to allow for nonlinear trends.

```
ct_model <- ctModel( #define the ctsem model
  manifestNames = c("State",'Behaviour'), #names of observed variables in dataset
  latentNames = c("StateDyn",'BehaviourDyn', 'StateTrend', 'BehaviourTrend'), #names of latent processes
  time = 'Time', #name of time column in dataset
  id = 'Subject', #name of subject column in dataset
  type='stanct', #use continuous time / differential equation model (standt for discrete-time / regression model)
  MANIFESTVAR = c('residualSD1',0,
    0, 'residualSD2'), #sd of the residual / measurement error
  LAMBDA = cbind(diag(1,2),diag(1,2)), #relating latent process to observed variables
  MANIFESTMEANS=0, #no measurement intercept / offset needed (1 observed variable relates directly to latent)
  CINT=c(0,0,'stateTrend||TRUE','behaviourtrend||TRUE'), #continuous intercept for trend, with random effects
  T0MEANS=c(0,0,'initialState||TRUE','initialBehaviour||TRUE'), #initial process values with random effects
  DRIFT = c('autoEffectS', 'crossEffectBS',0,0, #temporal effects 
    'crossEffectSB','autoEffectB',0,0,
    0,0,'statetrendDependence',0, #allow for nonlinearities in trend, otherwise no effect on trend process
    0,0,0,'behaviourtrendDependence'), #allow for nonlinearities in trend, otherwise no effect on trend process
  DIFFUSION = c('systemNoiseS', 0, 0, 0, #system noise matrix, 0 in upper triangle (because correlation only needs 1 par)
    'systemNoiseCross', 'systemNoiseB', 0, 0,
    0,0,0,0, #no system noise for trends
    0,0,0,0))
```

### 3.3 Fit and Summarise ctsem Model

We now fit the model to our data. By default, this uses maximum
likelihood. If you want to use priors for a Bayesian approach, this can
be requested by setting priors=TRUE. Please refer to the ctsem
documentation by running `ctDocs()` for more details.

```
ct_fit <- ctStanFit(datalong = data, ctstanmodel = ct_model) #fit the model to our data
```

```
## Error in delta[abs(delta) > maxparchange] <- maxparchange * sign(delta[abs(delta) >  : 
##   NAs interdits dans les affectations indicées
```

```
summary(ct_fit, parmatrices=FALSE) #print summary of the fit, some output disabled
```

```
## $residCovStd
##           State Behaviour
## State     0.375     0.084
## Behaviour 0.084     0.195
## 
## $resiCovStdNote
## [1] "Standardised covariance of residuals"
## 
## $rawpopcorr
##                                     mean     sd    2.5%     50%   97.5%       z
## initialBehaviour__initialState   -0.3077 0.1760 -0.4832 -0.4685 -0.1148 -1.7481
## stateTrend__initialState          0.4561 0.5614 -0.1395  0.9896  0.9946  0.8124
## behaviourtrend__initialState      0.2855 0.2311  0.0400  0.5043  0.5060  1.2354
## stateTrend__initialBehaviour     -0.1756 0.2649 -0.4440 -0.4076  0.1364 -0.6628
## behaviourtrend__initialBehaviour  0.1006 0.0216  0.0871  0.0981  0.1190  4.6545
## behaviourtrend__stateTrend        0.3579 0.2638  0.0822  0.5888  0.6222  1.3565
## 
## $popsd
##                     mean       sd   2.5%     50%    97.5%
## initialState      0.4749   0.1030 0.3063  0.4628   0.6981
## initialBehaviour  1.0070   0.1992 0.6626  0.9956   1.4493
## stateTrend       89.6679 125.8876 0.0001 14.0565 411.7259
## behaviourtrend    0.1455   0.0301 0.0954  0.1417   0.2110
## 
## $popmeans
##                                  mean       sd       2.5%      50%     97.5%
## initialState                   2.0105   1.0773    -0.1786   1.9995    4.1224
## initialBehaviour               4.2222   1.0174     2.1533   4.2304    6.1707
## autoEffectS                   -0.2981   0.0606    -0.4334  -0.2915   -0.1945
## crossEffectBS                  0.1220   0.1089    -0.0818   0.1225    0.3332
## crossEffectSB                  0.1552   0.1054    -0.0509   0.1558    0.3586
## autoEffectB                   -0.2949   0.0597    -0.4147  -0.2889   -0.1933
## statetrendDependence        -165.6101 227.4930  -748.0412 -33.5246    0.0000
## behaviourtrendDependence      -0.3108   0.0613    -0.4435  -0.3062   -0.2033
## systemNoiseS                   0.2140   0.0451     0.1390   0.2088    0.3192
## systemNoiseCross               0.0854   0.0538    -0.0196   0.0855    0.1942
## systemNoiseB                   0.2152   0.0457     0.1397   0.2098    0.3142
## residualSD1                    0.0723   0.0155     0.0468   0.0707    0.1068
## residualSD2                    0.0630   0.0133     0.0408   0.0616    0.0916
## T0var_StateDyn                 3.6731   0.5587     2.6278   3.6530    4.8203
## T0var_BehaviourDyn_StateDyn    0.1798   0.0498     0.0887   0.1819    0.2696
## T0var_BehaviourDyn             1.8379   0.3232     1.2944   1.8062    2.5384
## stateTrend                    92.2902 681.7733 -1190.4925  64.9856 1443.4693
## behaviourtrend                 1.1567   1.0815    -1.0042   1.1747    3.1642
## 
## $popNote
## [1] "popmeans are reported as specified in ctModel -- covariance related matrices are in sd / unconstrained correlation form -- see $parmatrices for simpler interpretations!"
## 
## $loglik
## [1] 318.21
## 
## $npars
## [1] 28
## 
## $aic
## [1] -580.4199
## 
## $logposterior
## [1] 318.21
## 
## $parmatNote
## [1] "For additional summary matrices, use argument: parmatrices = TRUE"
```

Note now the rawpopcorr section of summary shows the correlation
between behaviour and state trends, and the population mean for the
trends is reported in the popmeans.

### 3.4 Visualise Predictions

We can visualise predictions for the latent processes, independent of
data (so conditional only on estimated parameters), as follows:

```
ctKalman(fit = ct_fit, plot=TRUE, subjects = 3, kalmanvec=c('etaprior'), removeObs = T)
```

This allows us to see the direction and uncertainty associated with
the dynamic component, and the trend component, separately.
